# Systematic Review of Microorganism Removal Performance by Physiochemical Water Treatment Technologies

**DOI:** 10.1021/acs.est.4c03459

**Published:** 2025-03-28

**Authors:** Matthew Burke, Emma Wells, Caleb Larison, Gouthami Rao, Matthew James Bentley, Yarrow S. Linden, Patrick Smeets, Jennifer DeFrance, Joe Brown, Karl G. Linden

**Affiliations:** † 1877 University of Colorado Boulder, Boulder, Colorado 80303, United States; ‡ 41474 University of North Carolina Chapel Hill, Chapel Hill, North Carolina 27514, United States; § 126147 KWR Water Research, Groningenhaven 7, Nieuwegein, Utrecht 3430 BB, Netherlands; ∥ World Health Organization, Avenue Appia 20, 1211 Geneva, Switzerland; # Georgia Institute of Technology, Atlanta, Georgia 30332, United States

**Keywords:** World Health Organization, Log Reduction Value, LRV, Disinfection, Removal, Pathogen

## Abstract

Access to safe drinking water is crucial for public health necessitating the use of effective water treatment processes. We conducted a systematic literature review on microorganism removal by physical treatment processes used in drinking water treatment systems with the aim of providing current summary data to update the World Health Organization’s Guidelines for Drinking Water Quality (GDWQ) and to reflect on the data available for comparison of treatment technologies. We reviewed peer-reviewed articles reporting original data that were published between 1997 and March 2022 on the following physical treatment technologies: roughing filters, storage reservoirs, bank filtration, conventional and high-rate clarification, dissolved air flotation, lime softening, granular media filtration, slow sand filtration, precoat filtration, membrane filtration, granular activated carbon, ceramic membrane filtration, and soil aquifer treatment. The literature search was conducted in several databases including Web of Science and PubMed. Data from 165 articles were included in the analysis and used to calculate Log Reduction Values (LRVs) for each technology by microbial contaminant type (bacteria, virus, or protozoa). The quantity and quality of data ranged widely for each technology. We found granular media, membranes (microfiltration (MF), ultrafiltration (UF), and reverse osmosis (RO)), and precoat filtration to remove the most protozoa with average LRVs of 3.0 (95% CI 2.8–3.3), 5.7 (95% CI 5.4–6.0), and 4.4 (95% CI 4.1–4.7), respectively. Bacteria was removed most effectively by membrane filtration (MF, UF, RO) with average LRVs of 4.5 (95% CI 3.9–5.1) and moderately by dissolved air flotation, lime softening, and soil aquifer treatment with average LRVs of 2.7, 2.6, and 2.4 respectively. Viruses were removed most effectively by reverse osmosis membrane filtration with an average LRV of 4.9 (95% CI 4.0–5.7). This data provides valuable information on pathogen reduction and areas of needed research. The variation in results underscores the importance of further consideration when selecting technologies to use and the need for standardized reporting in both lab and field studies. It is important to consider variables in water quality and technology operation that may impact treatment effectiveness when selecting treatment options for use. The findings contribute to ongoing efforts to revise the WHO’s GDWQ, offering updated insights into LRVs for different water treatment technologies.

## Graphical abstract

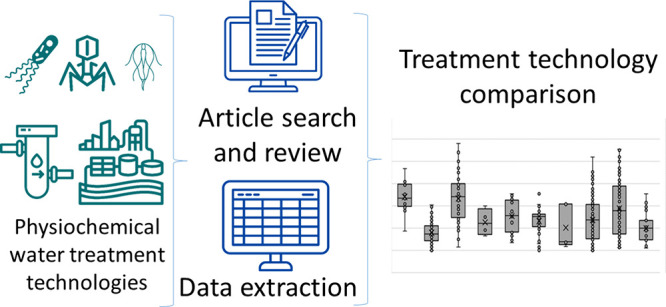

## Introduction

Safe drinking water is important for health and development, but natural water is rarely safe enough to drink without treatment. Surface water from rivers, lakes, and other fresh waterbodies and groundwater wells are some of the common sources of drinking water used around the world. These sources typically have some concentration of harmful biological contaminants in them, in the form of bacteria, protozoa, and viruses. These pathogens occur in water because of effluent discharge, combined sewer overflows, agricultural and urban runoff, wildlife, as well as open defecation, from humans and improper fecal waste management. Pathogens in drinking water can lead to waterborne illnesses and public health crises. As a result, treating water to remove or inactivate these pathogens before consumption has become a common practice. The World Health Organization (WHO) has published standards or guidelines on drinking-water quality since 1958 to help governments and water suppliers around the world in developing national standards and establish best management practices to ensure drinking water safety. As part of a multiple barrier approach to ensuring drinking-water safety, WHO includes guidance on water treatment. In the fourth edition of the *Guidelines for Drinking Water Quality* (*GDWQ*) published in 2011, the WHO included updated information and guidance on the effectiveness of water treatment processes in removing or inactivating microbial pathogens (bacteria, viruses, and protozoa), including a summary table on minimum and maximum removals for pathogens, as log reduction values (LRVs) that are achievable by water treatment technologies used in water treatment facilities. This information has been retained in the latest edition of the *GDWQ*, the fourth edition incorporating the first and second addenda, published in March 2022.^1^

This review is part of a broad effort to revise the WHO’s *GDWQ*, with a specific objective to review the LRVs for different water treatment technologies in more recent peer-reviewed literature to see how they can inform an update to the LRVs published by the WHO in its last publication of the *GDWQ.* The objective of this report is to review the LRVs of various methods of filtration (granular media filtration, precoat filtration, slow sand filtration, and membranes), methods of coagulation, flocculation and sedimentation (conventional clarification, high-rate clarification, dissolved air flotation, and lime softening), and methods of pretreatment (roughing filters, bank filtration, and storage reservoirs) included in the last publication of the *GDWQ* as well as three physical water treatment processes (filtration processes of granular activated carbon, ceramic membranes, and pretreatment using soil aquifer treatment) that are under consideration for inclusion in the WHO’s next edition of the *GDWQ*. While water treatment is vital for ensuring drinking-water safety, the most suitable technology is context specific and dependent on factors such as materials, ease of use, capital and operational costs, and source water quality. The LRV data presented in this literature review should be considered a starting point and local conditions taken into consideration when estimating achievable LRVs for monitoring and product evaluation purposes.

## Materials and Methods

A systematic literature review following the majority of the Preferred Reporting Items for Systematic Reviews and Meta-Analyses (PRISMA) best practices guidelines was undertaken to identify peer-reviewed journal articles examining the water treatment technology pathogen LRVs that have been published since the fourth edition of the *GDWQ.* ^2^ The criteria for a journal article to be included in the literature review included being published between 1997-March 22, 2022 and containing novel pathogen reduction data for water treatment. Journal articles referenced in the WHO’s 2004 publication of *Water Treatment and Pathogen Control*, which was the key WHO supporting reference for the GDWQ summary table were also included to capture data used from studies published prior to 1997.^3^ Reports from governments, funding agencies, nonpeer-reviewed conference proceedings and books were excluded. Articles that were literature reviews, focused on any type of water treatment besides small to large scale drinking water treatment, or did not present any new or original pathogen reduction data on bacteria, protozoa, or viruses were excluded from the analysis in this report.

Developing an effective search string was essential to the success of this literature review and the search was conducted in multiple steps. The first step was creating a large, inclusive master search string containing all the technologies that were being reviewed in the study. A copy of the master search string can be found in Supporting Information Table S1. This search string was applied in the databases Web of Science, PubMed, Scopus, Google Scholar, and AGRICOLA. After the master search, additional individual searches were conducted for each technology. An example search string for each individual technology can be found in Supporting Information Table S1. The individual search strings were used in Web of Science, PubMed, Google Scholar, as well as in Engineering Village and ScienceDirect, both of which had complications providing results from the full search string. All the articles from the master search string and individual search strings were evaluated to confirm that it was a study on water treatment and had original pathogen reduction data. In addition to the searches in databases since 1997, experts in the fields of specific drinking water treatment technologies were consulted individually and in virtual group meetings and asked to provide any articles they knew to be particularly important to include in our analysis, regardless of year. Experts also engaged in discussion to assess the risk of bias for studies included in this literature review.

Every article that was determined to be relevant to this review went through an extensive data extraction process. Articles were first categorized by the setting in which the study was performed. An efficacy or lab study was defined as an experiment that was performed under ideal conditions where there was considerable control over the variables and process of the experiment. Efficacy studies were typically undertaken in a lab setting, where spiked microbes can be added at higher densities, resulting in possibly greater LRV results compared to studies constrained by the input target concentration. Effectiveness or field studies were defined as an experiment that was conducted under real world conditions, and these studies typically took place at a pilot or active water treatment facility. The type, genus, species, strain, and the American Type Culture Collection (ATCC) code of the pathogen or surrogate evaluated in the article was recorded, although the strain and ATCC code was not reported in every article. Due to insufficient data or a lack of variation in the microorganisms studied, we analyzed data grouped by bacteria, viruses, or protozoa and were unable to analyze by specific species. Studies using culture-based assays, microscopy, or integrated cell culture/polymerase chain reaction (ICC/PCR) were included. Variations in rates of pathogen recovery by the methods included were not corrected for in the meta-analysis conducted in this article. Instead, it was assumed that recovery was addressed or accounted for in the data reported in each individual peer-reviewed article. The LRVs that were extracted from articles depended on how the information was presented in each report with prioritization given to the most “granular” data. When values were provided for individual pre- and post-treatment pairs this data was recorded. However, most articles provided summarized data (e.g., averages of experimental repeats) and therefore that data was included. Not every article reported statistical information and some articles only reported a LRV or percent reduction with no pre- and/or post-treatment pathogen concentrations.

Data presented in text or table format was prioritized over data presented in graphs when both were presented. The data that were presented in graphs was extracted using WebPlotDigitizer, an online program that analyzes images of graphs and extracts the underlying numerical data. Effect measures for each treatment technology were considered through reported LRV or a calculated LRV using eq 1 if pre- and post-treatment microbial counts were provided using arithmetic means as was agreed upon by experts and collaborators on this project. If a percent reduction in microbial concentrations was given, we calculated the pre- and post-treatment values using eq 2 and then used eq 1 to calculate the LRV:

Calculated LRV Using Pre- and Post-treatment Concentrations

$$\mathrm{LRV}=\log_{10}\frac{Mean\;Pr\mathrm{e‐}treatment\;microbial\;concentration}{Mean\;Pos\mathrm{t‐}treatment\;microbial\;concentration}$$ 1

Calculated Post-treatment Using Percent Reductions

$$Mean\;Pos\mathrm{t‐}treatment\;concentration=,Mean\;Pr\mathrm{e‐}treatment\;concentration\times \left(1-\left(\frac{\%Reduction}{100}\right)\right)$$ 2

In cases where we used eq 1 and the LRV was not provided directly in the peer-reviewed article, nondetects in post-treatment water were assumed to be 1 (per mL as colony/plaque forming unit), which provides a conservative estimate of the true LRV. The LRV calculations for non-detect estimates were included with other studies that had pre- and post-treatment microbial concentrations.

All relevant data were extracted into Microsoft Excel^4^ and analyzed using R version 4.1 ^5^ for arithmetic mean LRVs, 95% confidence intervals (CI), interquartile ranges (IQR), and data quality control such as standardizing variable names and formats. For technologies with more than ten data points, the 95% CI’s were included as a range of values that likely includes the population mean value with a 95% degree of confidence. The range of the 95% CI thus reflects the level of uncertainty of the mean based on available data.^6^

## Results

Using the search string in the Supporting Information Table S1, we identified 41,137 publications, with an additional 355 publications identified through experts. After duplicates were removed, 20,831 publications were screened, and 17,509 publications were deemed irrelevant for further review. We assessed 3,322 full text articles for eligibility and included 414 in the LRV analysis. It should be noted that the general search string and article selection processes included physical treatment technologies identified in this article and disinfectant technologies which are reported on in a forthcoming publication. For the physical processes reported herein, 165 articles were selected for data analysis. Supporting Information Figure S1 illustrates the screening process of articles, Supporting Information Table S2 contains the PRISMA checklist, and Supporting Information Table S3 contains the complete reference list of articles included in our analysis. The number of articles and data points used in this publication for analysis of physical processes for water treatment are presented in Table 1.

**Table 1:** Number of Journal Articles and Data Points Included in LRV Analysis

Technology	Pathogen Type	# Journal Articles	# Efficacy/Lab Studies	# Effectiveness/Field Studies	# Data Points
Roughing Filter	Bacteria	7	0	7	53
Storage Reservoirs	Bacteria	4	0	4	19
	Protozoa	4	0	4	12
	Virus	2	0	2	2
Bank Filtration	Bacteria	3	0	3	21
	Virus	1	0	1	9
Conventional Clarification	Bacteria	3	0	3	10
	Protozoa	7	2	5	44
	Virus	8	3	3	243
High-Rate Clarification	Protozoa	2	0	2	12
Dissolved Air Flotation	Bacteria	1	0	1	3
	Protozoa	7	4	3	60
	Virus	2	1	1	4
Lime Softening	Bacteria	1	1	0	15
	Protozoa	3	1	2	12
	Virus	1	1	0	5
Granular Media	Bacteria	10	4	6	61
	Protozoa	20	7	13	146
	Virus	6	4	2	45
Slow Sand Filtration	Bacteria	15	5	10	132
	Protozoa	7	3	4	65
	Virus	4	2	2	25
Precoat Filtration	Bacteria	3	2	1	73
	Protozoa	7	6	1	82
Microfiltration	Bacteria	1	1	0	7
	Protozoa	1	1	0	24
	Virus	9	8	1	44
Ultrafiltration	Bacteria	11	8	3	36
	Protozoa	2	0	2	19
	Virus	17	13	4	107
Nanofiltration	Bacteria	1	1	0	3
	Virus	1	1	0	1
Reverse Osmosis	Bacteria	5	4	1	11
	Protozoa	1	0	1	1
	Virus	11	6	5	15
Granular Activated Carbon	Bacteria	3	0	3	11
	Protozoa	2	0	2	10
	Virus	2	0	2	20
Ceramic Membrane	Virus	6	5	1	50
Soil Aquifer Treatment	Bacteria	13	0	13	21
	Protozoa	1	0	1	1
	Virus	18	0	18	92

## Technologies Included in GDWQ

### Pretreatment Technologies

#### Roughing Filters

Roughing filters are a method of media filtration that is used prior to application of other drinking water treatment processes. Roughing filters typically comprise one or more connected deep bed filters composed of granular media that decreases in size as water flows through the filter. The media that can be used may vary, with the main stipulations being the size of the media in each subunit. Roughing filters have been made of typical filtration materials such as gravel and quartz sand and novel resourceful materials such as broken burnt bricks, charcoal maize cobs, and broken stones from a quarry.^7^ Another design consideration of roughing filters is the direction of flow of water through the filter. Roughing filters can be designed to have water flowing horizontally, upward, or downward but horizontally flowing roughing filters appeared the most often in literature. One of the advantages of a roughing filter is it does not require external electrical energy for operation and maintenance.^7^ This coupled with the ease of using locally available materials makes roughing filters a preferred technology for water pretreatment in rural and low- to middle-income communities.

Even though roughing filters have primarily been used to reduce turbidity in water, studies have shown they can achieve some pathogen removal as well. Roughing filters can achieve a LRV of up to 2.2 for bacteria, with a mean of 0.9 (95% CI 0.8 to 1.0) calculated in this analysis, but removal has also been shown to be as low as 0.2.^8,9^ The effectiveness of roughing filters is impacted by the turbidity of the source water used as they are typically used for pretreatment.^8,9^ Despite the wide range in the literature, most studies indicated a typical LRV of 1.0 for bacteria in roughing filters.

#### Storage Reservoirs

Storage reservoirs are a desirable form of pretreatment because of the simplicity and ease of operation. Storage reservoirs are usually in the form of a natural lake, constructed reservoir, or engineered concrete storage tank. Constructed storage reservoirs are designed to hold at least 1 day of water supply, which can help water treatment facilities with flow stabilization if they have a water source with variable flow. The residence time of storage reservoirs varies greatly and depends on the type of reservoir. Natural lakes and constructed reservoirs can have residence times of multiple weeks, while engineered concrete storage tanks usually have a maximum residence time of three to 5 days. The lengthy residence times of storage reservoirs allows time for bacterial, viral, and protozoan pathogens to die off, although in some instances, pathogens can enter reservoirs from local animals and add to the pathogen burden.^10,11^ Increased storage time has been linked to higher LRVs,^12^ a trend that could be identified in Figure 1, but short circuiting through a reservoir could negatively affect the potential LRV and may be prevented through reservoir design or mixing.^13^ The LRVs for bacteria and protozoa average 1.5 (95% CI 1.0 to 2.0) and 1.7 (95% CI 1.2 to 2.1), respectively. In comparison the *GDWQ* ^1^ reports a minimum and maximum LRV of 0.7 to 2.2 for bacteria with a residence time greater than 40 days (approximately 6 weeks) and 1.4 to 2.3 for protozoa with a residence time of 160 days (approximately 23 weeks).

**Figure 1:**
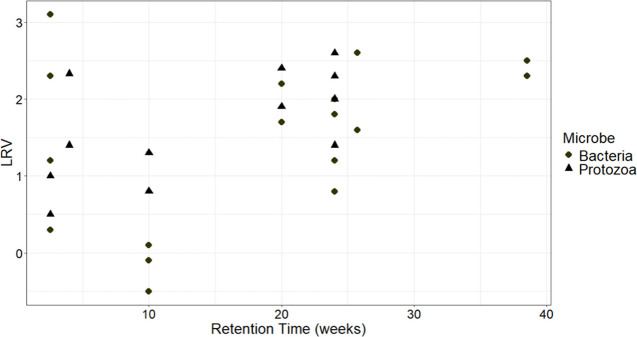
Bacteria and protozoa LRVs for storage reservoirs.

#### Bank Filtration

Bank filtration is a unique method of filtration that takes advantage of natural resources and processes. In bank filtration, a well is drilled in proximity of a river or lake and water is pumped from the well, drawing the surface water source through the subsurface, which is often diluted with native groundwater in transport. As the water is pulled toward the well, it is filtered through the subsurface sediment layers of the surface water banks. During the transportation from the surface water to the well, the water has time to undergo multiple physical, biological, and chemical processes. The water is physically filtered as it moves through sediments, and pathogens are further removed through adsorption, biodegradation, and redox reactions that occur naturally in the subsurface sediment layers.^14^ Bank filtration is commonly used in Northern Europe, but more sporadically around the world in low-, middle-, and high-income countries and is limited to communities located near a body of water.

The average LRVs for bacteria and viruses for travel distances of at least 20 m from source water to well inlet were 3.3 (95% CI 2.5 to 4.1) and 4.9 (insufficient data for CI), respectively (See full set of data in Figure S2 in the Supporting Information). Since bank filtration uses natural sediments to treat drinking water, and is a good, simple and robust barrier for surface water treatment, the travel distance and subsurface type are the main factors that need to be considered for this technology.^14^

### Coagulation, Flocculation, and Sedimentation Technologies

#### Conventional Clarification

Conventional clarification is a common component of drinking water treatment. Conventional clarification involves adding a coagulant to the water, rapidly mixing the coagulant into the water to promote the formation of larger particles and allowing these larger particles to settle out. The entire clarification process is typically carried out sequentially in a rapid mix basin, flocculation basin, and a sedimentation basin and the process is affected by pH, type of coagulant used (e.g., alum or ferric-based) and coagulant dose. The physical and chemical processes that occur during clarification require close monitoring by trained practitioners. The water chemistry (e.g., pH, turbidity, temperature, natural organic matter, etc.) in the raw water is likely to fluctuate, especially if it is from a highly variable source such as surface water. These fluctuations can require a change in coagulant dose to remain effective. The objective in clarification is focused on reducing turbidity and natural organic matter, but pathogens inevitably become adsorbed to these coagulated particles and settle out. After conventional clarification, an average LRV of 1.1 (95% CI 0.8 to 1.3) was calculated for protozoa, 1.1 (95% CI 0.8 to 1.4) for bacteria, and 1.6 (95% CI 1.5 to 1.7) for viruses.

#### High-Rate Clarification

High-rate clarification is an enhanced method of conventional clarification. In high-rate clarification, tubes or plates are placed inside the settling tank at a 45° to 60° angle depending on the size of particles settling. The presence of the angled tubes/plates in the settling basin decreases the settling distance of particles and decreases the overall volume of the settling tank. Studies have focused on high-rate clarification’s ability to remove protozoa, which is likely because of their large size relative to bacteria and viruses, which themselves can be captured in coagulated particles and settled out. For high-rate clarification an average LRV of 1.2 (95% CI 0.9 to 1.4) for *Cryptosporidium* and *Giardia* was calculated. Among these studies, high-rate clarification that used alum as a coagulant slightly outperformed high-rate clarification that used alum paired with a polymer by an average LRV of 0.2.^13^

#### Dissolved Air Flotation

Dissolved air flotation (DAF) is an alternative method of clarification used in drinking water treatment. Instead of allowing flocculated particles to settle out using gravity, as is done in traditional clarification processes, DAF uses release of pressurized air saturated water to atmospheric pressure to create bubbles that flocculated particles will attach to and rise to the surface of the water. The flocculated particles that rise to the surface are then removed by a desludging method (e.g., mechanical scraper). The clarified water exits through an outlet located at the bottom of the reactor. The coagulant dose used in DAF is important because it must ensure size of the flocs created can be efficiently removed through flotation.^15^ For example, Plummer et al. found the highest rates of *Cryptosporidium* removal corresponded to the highest dose (5 mg/L) of ferric chloride that they applied.^15^ DAF can remove pathogens such as *Cryptosporidium* and *Giardia* as they gain buoyancy when their flocs attach to rising bubbles.^15^ Based on the DAF literature reviewed, the average LRV for *Cryptosporidium* and *Giardia* was 2.4 (95% CI 2.2 to 2.6). There were minimal data on reduction of bacteria and viruses which had average LRVs of 2.7 and 2.5, respectively. As with most technologies that use coagulation, the most common coagulants used for DAF in the literature were alum and ferric chloride. The LRVs for these protozoa were not greatly impacted by the type of coagulant. Use of alum resulted in an average LRV of 2.4 while use of ferric chloride resulted in an average of 2.6.

#### Lime Softening

Lime softening is a technology that is typically used in water treatment facilities that use source water with high hardness (containing high levels of dissolved minerals). Hard water can cause operational issues from the formation of scales in pipes. In the lime softening process, lime in the form of calcium hydroxide is added to raise the pH of water to approximately 10.5, which allows particles to precipitate and settle out, and coagulants are added to ensure small minerals settle out. After the minerals settle out, the water is then recarbonated and the pH is lowered back to the levels before the lime softening process. Depending on the type of hardness present, lime softening is completed in one or two stages. Single stage lime softening is used when calcium is the main mineral present, and two stage lime softening is used when magnesium is present in the water. In addition to controlling mineral content, lime softening can be a useful tool for removing pathogens in water treatment facilities. During the lime softening process, pathogens are removed from the water through enmeshment with or aggregation within flocculated minerals and coprecipitation.^16^ Our analysis showed an average LRV of 2.6 (95% CI 1.9 to 3.3) for bacteria, 2.0 (insufficient data for CI) for viruses, and 1.1 (95% CI 0.5 to 1.6) for protozoa.

### Filtration Technologies

#### Granular Media Filtration

Granular media filtration is one of the most common forms of filtration in drinking water treatment. Granular media filtration is used across the globe and can easily be scaled to serve large populations or small communities because of its simplicity to operate and wide availability of materials for filter media. Granular media filters are designed to use single or dual media and typically use silica sand, anthracite, or some combination of both. In media filtration, water flows through the densely packed granular media and microbes are removed through physical straining or from adsorption, interception, or sedimentation onto the granular media following chemical destabilization. Due to the physio-chemical nature of granular filtration’s removal mechanism, coagulation is a critical factor in removing pathogens from the water because it creates destabilized and larger microbe-associated particles that are easier for the granular media to intercept from the water.

Our analysis found the typical log reduction for pathogens ranges from 1.5 to 3.0, depending on whether the target pathogen is bacteria, protozoa, or viruses, and will vary with filter properties such as grain size and media type. Figure 2 shows the full range of LRVs found for granular media filtration from the identified studies. The LRV for protozoa can be increased to approximately 1.5 with optimal coagulation, compared to a LRV of approximately 0.5 without coagulation.^17^ The log reduction of bacteria in granular filtration can range between 1 to 3.5 (mean of 1.8 with 95% CI of 1.5 to 2.1) and the reduction of protozoa can range from 0.3 all the way up to 6.6 (mean of 3 with 95% CI of 2.8 to 3.3). Despite the number of studies on granular filtration, it is difficult to assign a single log reduction value to bacteria, protozoa, and viruses because of the variability in filter operations. The source water, pretreatment processes, as well as coagulant type and dose, all impact the LRV proficiency of granular filtration.

**Figure 2:**
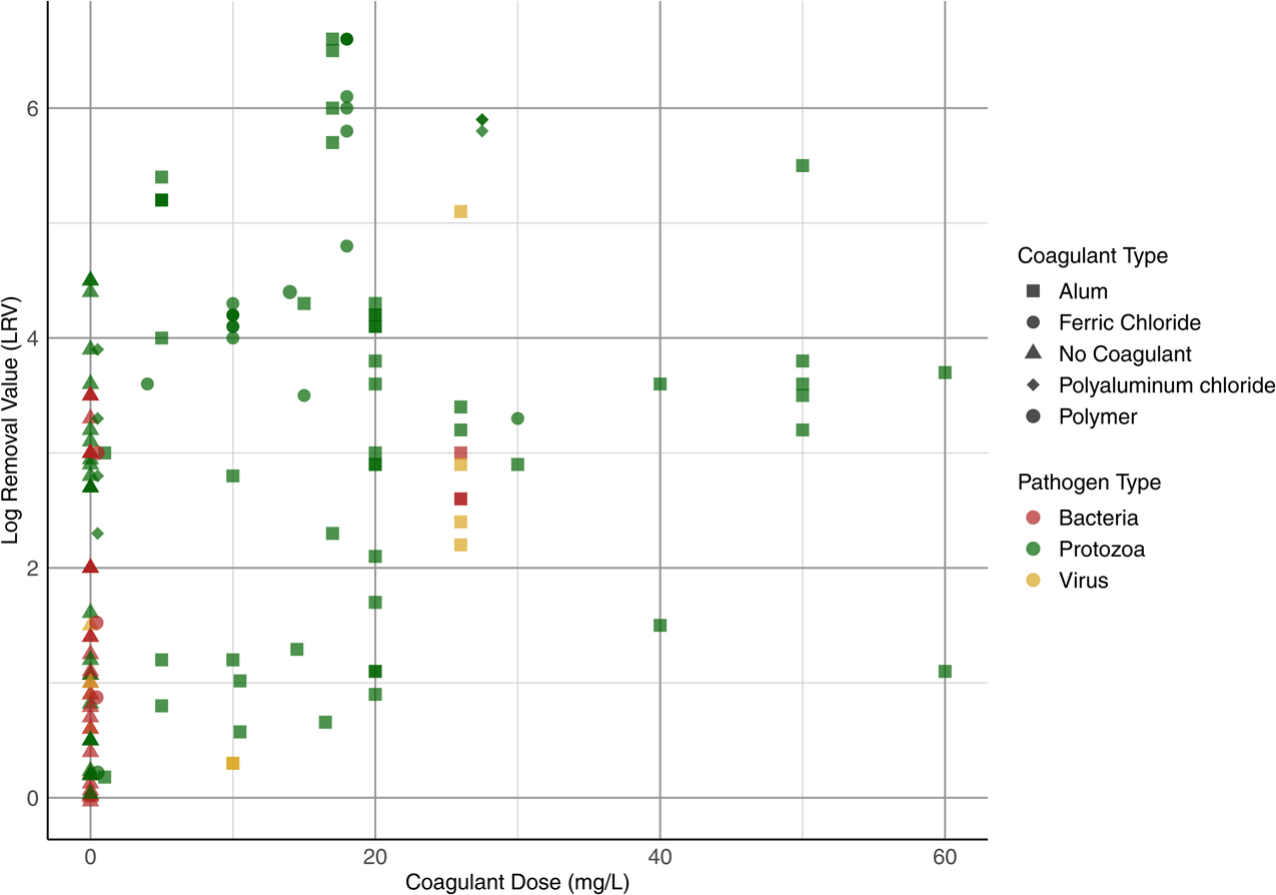
Pathogen LRVs found for granular media filtration.

#### Slow Sand Filtration

Slow sand filtration is one of the oldest technologies in drinking water treatment. A slow sand filter consists of fine filter media, typically sand, and a synthetic support layer. Water flows slowly through the sand filter and pathogens are removed through biological and physical processes. Large pathogens are physically removed by straining as water flows through the slow sand filter. A biological layer called the schmutzdecke develops at the top of the slow sand filter during normal filter operation. Once formed, the schmutzdecke matrix of microorganisms can biologically degrade pathogens through predation, leading to pathogen inactivation in slow sand filtration.^12^ As the schmutzdecke develops and grows in the filters, the filter resistance increases, leading to a thick water layer on top of the filter, increasing the water volume and residence time. In order to keep the filter in optimal performance, the schmutzdecke needs to be periodically (every 1 to 12 months or longer) partially removed from the filter by a manual or mechanical scraper. Most slow sand filters are operated without pretreatment and/or chemical coagulation and are sometimes operated as the last stage in water treatment. The simplistic design and operational ease make slow sand filtration a valuable technology, however it has low filtration rates, which lead to large surface areas required for treatment.

*Giardia* was the most frequent protozoa pathogen used in the studies found for this literature review and demonstrated a fairly large range of LRVs with a minimum LRV of 0.31 and a maximum LRV of 4.4. The total average LRV was 2.6 (95% CI 2.4 to 2.9) for all protozoa. It is imperative that slow sand filtration is also effective at removing bacteria and viruses since it is typically used in a treatment train with fewer barriers for pathogen removal than conventional treatment. Total coliforms, while not an ideal indicator for bacterial removal performance due to its potential to grow in the filter, appeared most often in studies on slow sand filtration. The range of LRVs found for total coliforms was smaller than the range of LRVs found for *Giardia.* The minimum LRV found for total coliforms was 0.6 and the maximum LRV found was 3.4. The average LRV for all bacteria was 1.7 (95% CI 1.6–1.9). Recent studies have found that slow sand filtration can achieve modest LRVs for viruses with an average LRV for all viruses of 2 (95% CI 1.4 to 2.5). Bacteriophage MS2 was used most as an indicator of virus removal in slow sand filtration and achieved a LRV ranging from 0.2 to 2.2.^12^ The source water, filter media characteristics, and the state of the schmutzdecke all have some variations which impact the LRV proficiency of slow sand filtration. While no correlation of removal with the schmutzdecke age was analyzed, it is common practice following scraping of the schmutzdecke during slow sand filter cleaning to use a ripening period of one to a few weeks before restarting filtration.^3^

#### Precoat Filtration

Precoat filtration was initially developed as a method of mobile water treatment by the United States military for protozoan parasites, although it is occasionally used in drinking water treatment facilities today. Precoat filtration uses pressure or a vacuum to filter water through a uniformly thin layer of filter media located on a permeable support structure, called the precoat. The filter media, typically a naturally occurring mineral such as diatomaceous earth or perlite, is added to the filter feed as a slurry. Once added to the filter feed, the filter media in the slurry mixture can remove contaminants from the water through straining and deposition. The slurry mixture and pathogens in the water that were not adsorbed by the slurry are then mechanically strained by the pressure driven filtration through the precoat. During the operation of the precoat filter, a filter cake forms on the support structure containing the removed microbes, particles, and filter media from the slurry mixture. The filter cakes must be periodically removed from the filter for it to maintain its proficiency. Given precoat filtration requires constant pressure or vacuum for its operation, this technology is limited to areas that have a continuous and reliable source of energy.

Precoat filtration has been shown to have high LRVs for protozoa, with studies reporting a LRV as high as 6.7 for *Cryptosporidium.* ^18^ The average LRV for all protozoa was 4.4 (95% CI 4.1 to 4.7) and 1.3 (95% CI 1.2 to 1.5) for all bacteria. Because the removal mechanism is mainly physical, turbidity levels of source water, filtration rate, and filter media type greatly impact the microbial LRV of precoat filtration technology. Ongerth and Hutton found higher filter grade material and higher flow rates through filters increased the removal of *Cryptosporidium.* ^18^ The removal mechanism for precoat filtration is primarily physical and does not require chemical coagulation like granular media filtration, which reduces the operational costs and variability of precoat filtration.

#### Membranes

Membranes used in drinking water treatment encompass four different processes: microfiltration (MF), ultrafiltration (UF), nanofiltration (NF), and reverse osmosis (RO). The principal difference in each technology is the size of the membrane pores, which are typically defined as microfiltration (∼0.1 μm), ultrafiltration (∼0.01 μm), nanofiltration (∼0.001 μm), and reverse osmosis (∼0.0001 μm), along with differences in membrane materials. In all cases, these definitions were used when categorizing membrane filtration technologies. Filtration membranes are growing in use at drinking water treatment facilities in high income countries because of the excellent removal of protozoa and viruses that can be achieved with MF and UF, respectively. Pathogens are removed by forcing water through the small pores of the membrane, leaving behind the pathogens. Thus, membranes are a pressurized system that require a constant source of power for its operation. Due to the amount of pressure needed to operate, MF and UF are considered low pressure membranes (<30 psi) while NF and RO are considered high pressure membranes (>75 psi). Membrane processes are also sensitive to turbidity and natural organic matter in the source water. High levels of turbidity and natural organic matter lead to reversible or irreversible fouling, that reduces the effectiveness of the membrane and may require chemical cleaning. This high sensitivity makes pretreatment vital if membranes are utilized in a drinking water treatment process.

The rule of thumb in drinking water treatment is MF is implemented to remove bacteria and protozoa, but viruses are small enough to flow through the 0.1 μm pores. However, studies have shown that MF membranes can achieve some removal of viruses, likely due to adsorptive interactions between virus particles and the membrane surface and removal by solids previously deposited on the membrane surface.^19^ The mean LRV for viruses in MF was 1.8 (95% CI 1.3–2.3). The literature on UF was similarly focused on the removal of viruses. The mean LRV for viruses in UF was 2.8 (95% CI 2.5 to 3.2), although a large range of LRVs were reported and studies covered several types of viruses. Allolevivirus and bacteriophage GA has the lowest LRVs among viruses in UF, with reported LRVs as low as 0.0005 and 0.1, respectively.^20^ One study demonstrated a LRV of 8.2 for bacteriophage MS2 which is the highest LRV for viruses found for UF.^21^ The average LRV for bacteria with UF was 4.6 (95% CI 3.7–5.5), but there was a large range in these data as well. For RO, the LRVs found for bacteria were from data representing heterotrophic plate counts (which could be skewed due to regrowth in the permeate) and ranged from 1.2 to 6.3 while the LRV for viruses ranged from 1.0 to 7.0. The large ranges of LRVs for each membrane technology conveys the difficulty of assigning a single LRV for pathogens to a technology. Figure 3 and Table 2 show log reductions for bacteria, protozoa and viruses broken out by membrane type. Based on these data, it is clear that removal of bacteria and protozoa are relatively similar across all membrane types as would be expected based on the size of the organisms, with the exception of data on NF, which was limited. The ultimate log removals are dependent on the influent concentrations, as well as the integrity of membrane materials and seals, as well as factors related to membrane materials that may affect adsorption and electrostatic repulsion. Viruses however appear to be removed more differentially with MF being the poorest at removal and RO being the most effective at removal, as expected with decreasing pore size, although additional data on NF performance is needed. Beyond membrane type and pore size, the virus size and stock used in studies may influence the LRV achieved as virus size can vary and viral stocks may clump when prepared in the laboratory.^20^

**Figure 3:**
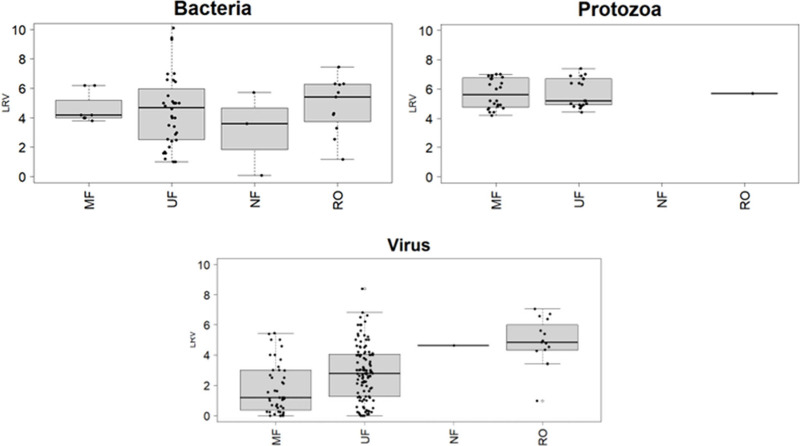
Pathogen LRVs for each membrane type. Each box and whisker plot represents the first to the third quantiles with the line indicating the median value. The whiskers show the minimum and maximum values, exclusing outliers. MF = Microfiltration, UF = Ultrafiltration, NF = Nanofiltration, and RO = Reverse Osmosis.

**Table 2:** LRVs from the WHO’s GDWQ (2022) Compared to LRVs from This Analysis

		LRVs in Current Edition of the GDWQ		Mean LRVs and 95% Confidence Intervals around the Mean, Found in This Study			First and Third Quartiles and Median LRVs Found in This Study		
Technology	Pathogen Type	Minimum	Maximum	Lower 95% C.I.	Average	Upper 95% C.I.	25th	Median (50th)	75th
Roughing Filter	Bacteria	0.2	2.3	0.8	0.9	1.0	0.5	0.8	1.2
Storage Reservoirs	Bacteria	0.7	2.2	1.0	1.5	2.0	1.0	1.7	2.3
	Protozoa	1.4	2.3	1.2	0.7	2.1	1.2	1.7	2.3
Bank Filtration	Bacteria	2.0	>6.0	1.2	1.7	2.2	1.5	3.7	4.7
	Protozoa	>1.0	>2.0	^a^	^a^	^a^	^a^	^a^	^a^
	Virus	>2.1	8.3	^a^	2.9	^a^	4.0	5.0	5.9
Conventional Clarification	Bacteria	0.2	2.0	0.8	1.1	1.4	0.7	1.1	1.3
	Protozoa	1.0	2.0	0.8	1.1	1.3	0.6	0.8	1.3
	Virus	0.1	3.4	1.5	1.6	1.7	0.9	1.6	1.3
High-Rate Clarification	Protozoa	>2.0	2.8	0.9	1.2	1.4	0.8	1.1	1.6
Dissolved Air Flotation	Bacteria	-	-	^a^	2.7	^a^	^a^	2.5	^a^
	Protozoa	0.6	2.6	2.2	2.4	2.6	2.0	2.5	2.9
	Virus	-	-	^a^	2.5	^a^	1.7	2.4	3.3
Lime Softening	Bacteria	1.0	4.0	1.9	2.6	3.3	2.1	2.7	3.7
	Protozoa	0.0	2.0	0.5	1.1	1.6	0.6	0.9	3.2
	Virus	2.0	4.0	-^a^	2.0	^a^	0.7	0.8	4.2
Granular Media Filtration	Bacteria	0.2	4.4	1.5	1.8	2.1	1.0	2.0	2.7
	Protozoa	0.4	3.3	2.8	3.0	3.3	1.7	3.0	4.2
	Virus	0.0	3.5	2.3	2.6	3.0	2.2	3.0	3.2
Slow Sand Filter	Bacteria	2.0	6.0	1.6	1.7	1.9	1.0	1.6	2.3
	Protozoa	0.3	>5.0	2.4	2.6	2.9	1.9	3.0	3.3
	Virus	0.3	4.0	1.4	2.0	2.5	1.0	2.0	2.7
Precoat Filtration	Bacteria	0.2	2.3	1.2	1.3	1.5	0.8	1.3	1.7
	Protozoa	3.0	6.7	4.1	4.4	4.7	3.0	4.7	5.7
	Virus	1.0	1.7	^a^	^a^	^a^	^a^		^a^
Membrane Filtration (MF, UF, NF, RO combined)	Bacteria	1.0	>7.0	3.9	4.5	5.1	2.9	4.3	6.1
	Protozoa	2.3	>7.0	5.4	5.7	6.0	4.9	5.5	6.7
	Virus	<1.0	>6.5	2.5	2.8	3.1	1.1	2.7	4.2
Microfiltration (MF)	Bacteria	-	-	^a^	4.7	^a^	4.0	4.2	5.2
	Protozoa	-	-	5.3	5.7	6.1	4.8	5.6	6.7
	Virus	-	-	1.3	1.8	2.3	0.4	1.2	3.0
Ultrafiltration (UF)	Bacteria	-	-	3.7	4.6	5.5	2.5	4.7	5.7
	Protozoa	-	-	5.3	5.8	6.2	4.9	5.2	6.7
	Virus	-	-	2.5	2.7	3.2	1.3	2.8	4.1
Nanofiltration (NF)	Bacteria	-	-	^a^	3.1	^a^	^a^	3.6	^a^
	Virus	-	-	^a^	4.6	^a^	^a^	4.6	^a^
Reverse Osmosis (RO)	Bacteria	-	-	3.5	4.8	6.0	3.8	5.4	6.3
	Protozoa	-	-	^a^	5.7	^a^	^a^	5.7	^a^
	Virus	-	-	4.0	4.9	5.7	4.3	4.8	6.0
GAC	Bacteria	-	-	0.2	0.6	1.1	0.3	0.5	0.6
	Protozoa	-	-	0.9	1.5	2.1	1.4	1.6	2.0
	Virus	-	-	2.4	3.1	3.8	1.8	3.1	4.5
Ceramic Membrane	Bacteria	-	-	^a^	^a^	^a^	^a^	^a^	^a^
	Protozoa	-	-	^a^	^a^	^a^	^a^	^a^	^a^
	Virus	-	-	4.1	4.7	5.3	3.0	4.8	6.0
SAT	Bacteria	-	-	1.5	2.4	3.4	0.9	1.9	4.0
	Protozoa	-	-	^a^	0.4	^a^	^a^	0.4	^a^
	Virus	-	-	3.8	4.3	4.8	2.4	4.0	5.9

a 10 or fewer data points.

## Potential Technologies to Include in the *GDWQ*

Granular activated carbon, ceramic membranes and soil aquifer treatment are not included in the WHO’s *GDWQ* microbial treatment Table 1. However, these technologies are increasingly being used in drinking-water treatment facilities around the world and therefore efficacy and effectiveness data related to these technologies warrant being reviewed.

### Granular Activated Carbon

Granular activated carbon (GAC) is pyrolyzed carbon commonly made from bituminous coal, peat, wood, and coconut shells. Each form of carbon stock generates GAC with slightly different characteristics, with the biggest variations being in the porosity and surface area of the GAC. Drinking water treatment plants apply GAC in several ways; GAC is used in media filters along with other types of granular media or it can be used as the fixed bed media in adsorption filters.^22^ While GAC is already widely used in drinking water treatment facilities around the world to help treat waters containing high natural organic matter and chemical pollutants, GAC also possesses the ability to help remove pathogens through filtration and adsorption.^22^ We calculated that GAC can achieve an average LRV of 0.6 (95% CI 0.2 to 1.1) for bacteria and 3.1 (95% CI 2.4 to 3.8) for viruses, and 1.5 (95% CI 0.9 to 2.1) for protozoa.

### Ceramic Membranes

Ceramic membranes are a type of membrane filter that typically have a porosity similar to MF or UF. The pore sizes in ceramic membranes are not highly uniform and are typically in the range of MF and UF, however it can have pores that are comparable to NF as well. Ceramic membranes differ from polymeric membrane technologies because they utilize a porous layer of inorganic compounds such as aluminum oxide, titanium oxide, or silicon carbide instead of polymers. The literature reviewed did not include ceramic membranes infused with metals such as silver. Given the wide range of pore sizes available for ceramic membranes, they can potentially be an effective treatment for bacteria, protozoa, and viruses. Werner et al. found that pore size had a significant impact on the LRV achieved by ceramic membranes with highest removal achieved by the smallest pore size.^23^ Based on the literature reviewed on ceramic membranes with pore sizes similar to UF, the average LRV for viruses was 4.7 (95% CI 4.1–5.3).

### Soil Aquifer Treatment

Soil aquifer treatment (SAT) is a form of artificial aquifer recharge. In SAT, water is applied to the land and allowed to infiltrate natural sediments and join the unconfined aquifer. In recent years, SAT has been used as a method of indirect potable reuse of municipal wastewater. Similar to bank filtration, SAT allows the soil to treat the water through a variety of natural processes including physically filtering the water, adsorption onto sediment particles, and biodegradation.^24^

The studies on the LRV potential of SAT primarily explored its proficiency at removing bacteria and viruses from water. *E. coli* and total coliforms were frequently used in studies to measure the proficiency of SAT at inactivating/removing bacteria while somatic and F-specific coliphages were frequently used in studies to measure the proficiency of SAT at inactivating/removing viruses. There was a wide range of LRVs found in studies of bacteria with LRVs over a range of approximately 0.7 to 7.5, which is due to differences in the soil characteristics, depth of the vadose zone and other variables that can have a huge influence on pathogen removal efficiency. The range of LRVs was even greater for viruses ranging from 0.26 to 9.1. Overall, it was determined that SAT had an average LRV of 2.4 (95% CI 1.5 to 3.4) for all bacteria and an average LRV of 4.3 (95% CI 3.8–4.8) for all viruses.

## Discussion

The data sets extracted for each technology’s respective pathogen LRVs indicated a wide range of treatment effectiveness, as can be seen below in Figure 4. In Figure 4, each box and whisker plot denote the average LRV calculated in this analysis, with the top of the box representing the 75th percentile of the data and bottom the 25th percentile of the data and 95% confidence interval of the mean represented by the whiskers. The average LRVs for each technology are representative of the data sets available in the literature but they may not truly be reflective of the removal ability each technology is capable of under proper operating conditions, as illustrated by the range of LRVs for each technology and pathogen class. The 75th percentile value of the LRVs reported herein further reflect the distribution of data available and may be considered the upper “potential LRVs” for system design goals when planning a well operated treatment system around relevant parameters (membrane manufacturer, size of filtration material, soil characteristics, residence time, etc.). Likewise, the 25th percentile LRVs may be considered the lower potential LRVs of the distribution of available data and could be used as conservative LRV values for when systems are not well maintained, or conditions are at risk or unknown. As such, it is essential that LRVs are considered in the context of local conditions including source water quality and operations as well as system design and selected technology providers. These data can be used as a resource to compare the effectiveness of treatment technologies across different pathogen classes and provide an initial representation of expected log reductions, that may be adjusted based on local conditions, expert judgment and additional data sources.

**Figure 4:**
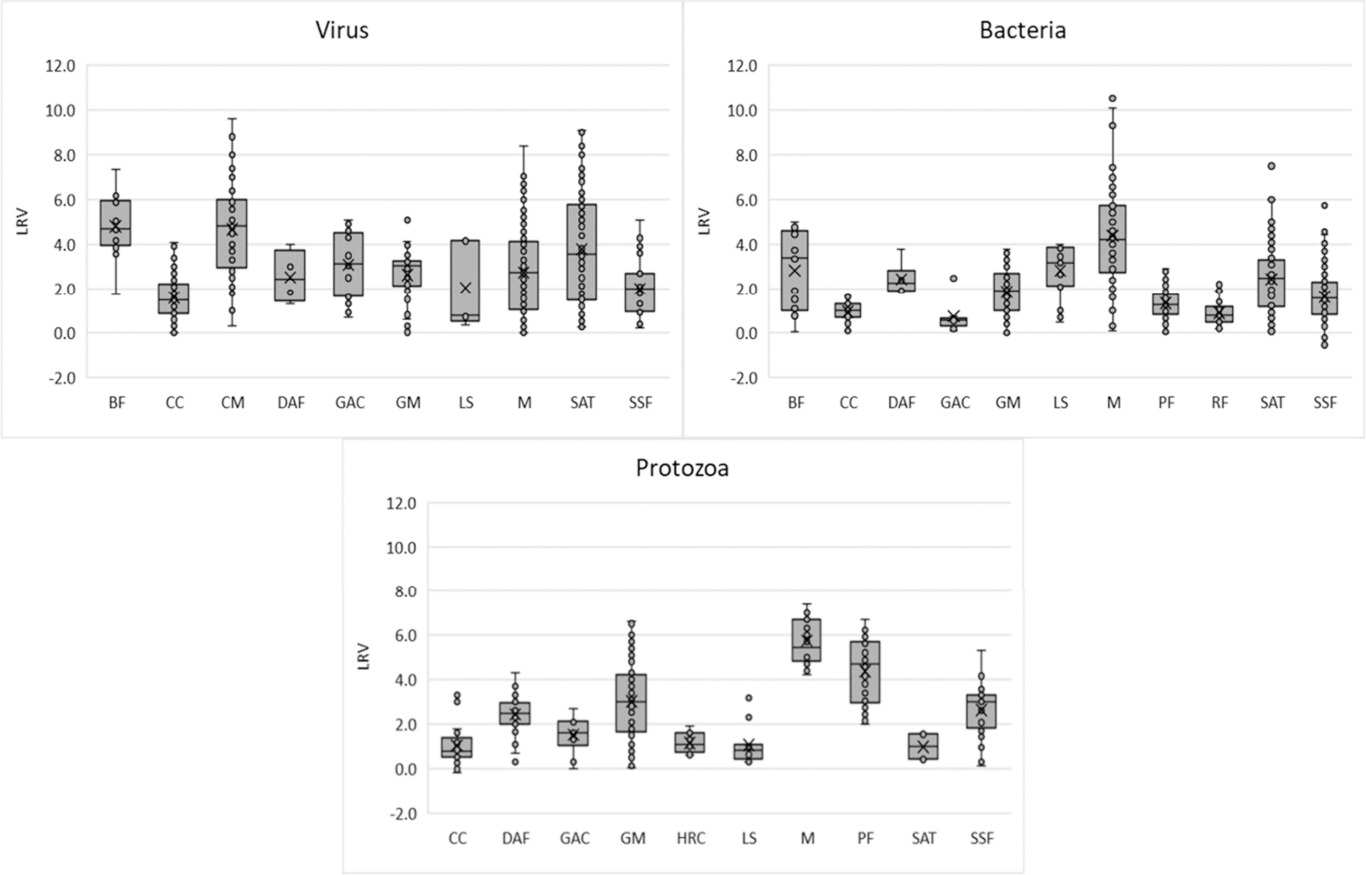
Box and whisker plots for pathogen LRVs for each technology reviewed. Each box represents the first to the third quantiles with the line indicating the median value. The X indicates the mean of the data. The whiskers show the minimum and maximum values, excluding outliers. RF = Roughing Filtration, BF = Bank Filtration, CC = Conventional Clarification, CM = Ceramic Membrane, DAF = Dissolved Air Flotation, GAC = Granular Activated Carbon, GM = Granular Media Filtration, HRC = High-rate Clarification, LS = Lime Softening, M = Membranes (Microfiltration, Ultrafiltration, Nanofiltration, Reverse Osmosis; see Figure 3 for data disaggregated by membrane type), PF = Precoat Filtration, SAT = Soil Aquifer Treatment, SSF = Slow Sand Filtration, SR = Storage Reservoir.

The fourth edition of the GDWQ^1^ published in 2022 presents minimum and maximum LRVs for the various technologies and much like the results presented in this study, there is a wide range between the possible minimum and maximum LRVs for a specific technology (See Supporting Information Table S4). Compared to this study, the minimum and maximum values provided in the GDWQ were determined based on more limited data sources, as well as expert judgment. In this study, the 95% confidence interval around the mean indicates the uncertainty of this mean value based on the available data. The average LRVs with their corresponding 95% confidence interval found as a result of this literature review are summarized below in Table 2, representing the summary of all the data compiled in this review. Table 2 reports the 25th and 75th percentiles of the LRVs calculated for each technology to reflect the distribution of the data available in the literature and for use as potential minimum and maximum LRV values for each technology.

The performance of each technology is sensitive to a variety of factors. Source water quality and flow rate can vary dramatically, changing the effectiveness of a technology in a matter of days, weeks or months. For these reasons, selecting a treatment sequence tailored to a water source is vital. Not only is it important that the correct technologies and pretreatment options are considered, but it is critical to have trained engineers and practitioners on hand to change operational parameters (e.g., flow rate, coagulant doses, etc.) to accommodate any subtle or drastic changes in the source water that could impact the effectiveness of each water treatment technology to reduce pathogens. Considering these aspects and given lab studies usually are conducted under ideal conditions, the data has been further presented, based on findings from lab studies, field studies and a pooled analysis in Supporting Information Tables S5. For technologies where there were both lab and field data, unsurprisingly, higher average LRVs were calculated based on lab studies for several technologies. However, in some cases, higher LRVs were calculated when considering field data only (e.g., for bacteria and protozoa, lime softening and granular media filtration, and for viruses, conventional clarification and dissolved air flotation, although aside from granular media filtration, lab data were much more limited for these technologies).

The ranges in LRVs for each technology indicate that the reported values in this study should be used with care. Mean LRVs and the 25th and 75th percentiles of available data provide a starting point for estimating total treatment performance when little is known about the actual practical conditions. When evaluating treatment systems for pathogen removal, the actual design, operating conditions, water quality and performance based on measurements of microorganisms or proxies (e.g., turbidity after filtration) need to be considered and can lead to choosing either lower or higher expected LRVs.^25,26^ Higher LRVs for existing systems could be verified by monitoring water quality at each stage of treatment. When systems were designed or operated suboptimally, or potential hydraulic issues (inefficient mixing of disinfectants, potential short circuits) are suspected, lower range LRVs should be chosen. When designing a system, attention can be paid to these issues, making higher range LRVs more likely, thus, overdesigning a system and unnecessary use of resources can be prevented. In all cases, validation and/or verification of performance in practice is essential to achieve safe drinking water.

## Limitations

The findings from this this systematic review should be considered alongside the limitations. The major limitations are

- Literature was limited for several technologies and did not equally represent performance in lab and field settings. This may be due in part to the limited date ranges used for this literature review. Further, several technologies examined are considered well established, including as part of conventional treatment and presumably is the reason for limited studies identified in the more recent literature. In addition, some technologies may not have been assessed, or assessed less, for a pathogen class because the outcome of such testing could be straightforwardly induced (e.g., a filter relying on mechanical filtration might not test for protozoan reduction if it were shown to be effective for bacterial reduction based on size) or because there are known limitations of the technology or method that makes specific testing uninformative with respect to performance. Lastly, we limited our search to published peer-reviewed journal articles and thus data from potentially other reputable publications (e.g., government reports, books) were excluded.
- Data quality and information regarding methods and study characteristics varied widely between studies. This limited our ability to provide meaningful analysis of how research methods and other factors such as water quality characteristics, design and operational conditions of technologies (e.g., flow rate and media size) may have influenced the results of our analysis. While several papers studied variations of these factors, all their data were included. For implementation of these recommendations, an engineer would choose the appropriate conditions (e.g., residence time of SAT) to achieve a high LRV in a design of a system.
- The arithmetic mean LRVs reported are impacted by extreme LRV values extracted from included journal articles; reported LRVs that may be uncharacteristic of the technology performance skew the average LRV calculations. The use of arithmetic means of LRVs as introduced by Hijnen and Medema has been debated.^27^ Schmidt et al. propose that LRVs be summarized by averaging reduction and then presenting this as a log value which they refer to as the effective log-reduction.^25^ Smeets et al.^26^ argue that weighted-average LRVs are an appropriate starting point in analysis that should be verified along with an assessment of variability and uncertainty of site-specific conditions. Given the variability of data available for analysis, we utilized or calculated the arithmetic mean LRVs from our selected studies while also providing the 95% confidence interval around the mean and the interquartile ranges (25th and 75th percentiles). To enable further analysis of this data, the full data set is available on Open Science Framework at https://osf.io/d2hax/..
- In many studies, the reported LRV was constrained by the lower limit of detection (LLOD). A LLOD is common in studies that do not include an artificial spike of pathogens before water treatment and occurs when the pathogen in treated water is not detectable. In this scenario, the reported LRV may actually be higher because the technology successfully removed all detectable pathogens in the sample. Furthermore, the LLOD limited LRVs can lower the average technology LRV that was calculated in this analysis.
- As highlighted by our panel of experts, our findings may be susceptible to publication bias, potentially skewing the systematic review’s representation of actual performance because many articles published include of novel, unexpected (e.g., outlier), or selectively “positive” results. It is important to note that we did not quantitatively evaluate these biases during the review process and did not measure uncertainty beyond the 95% confidence intervals. However, we still believe the overall quality of these LRVs refines what has been previously published and utilized in guidance documents. These data should be considered a starting point and local conditions taken into consideration when estimating achievable LRVs for monitoring and product evaluation purposes.

## Recommendations

The following major recommendations were reached as a result of this literature review:

- The next edition of the *GDWQ* should continue to provide a range in pathogen LRVs for water treatment technologies The interquartile range can be used to understand the distribution of the data available and may provide insight into the expected range of LRVs that each technology may provide depending on operating conditions. These results should be considered in the context of the data set used. Therefore, reported herein is the mean, median, confidence intervals, interquartile ranges, and the number of data points supporting these analyses.
- Current WHO LRV guidance combines all membrane technologies into one category, but it is recommended to include subcategories for LRVs of specific membrane technologies. As shown in Figure 3, each type of membrane provides a different level of treatment, and this would be better reflected if the membranes were separated instead of combined into one generic category.
- Further consideration should be made to including GAC, ceramic membranes, and SAT in the treatment table for the next edition of the *GDWQ.* There is sufficient data that supports including SAT as a reliable form of reducing pathogens as a pretreatment technology, however more data may be needed to support the inclusion of GAC and ceramic membranes. Including these technologies in the next edition of the *GDWQ* will provide better guidance for engineers and practitioners who are currently operating the technologies as well as provide more options for engineers, regulators, and policymakers to examine when considering new drinking water treatment options.
- Considering the variable quality and quantity of data available for several technologies in this systematic review, these data may not fully capture log reduction values expected from each technology. We recommend that our results be reviewed and considered alongside expert opinion and other reputable data sources, to inform the update of LRVs presented in the next GDWQ treatment table.
- The updated LRV treatment tables in the GDWQ, particularly if average values are presented, should be accompanied with text encouraging localized assessment of source water quality when considering different water treatment options, to consider appropriate process conditions in the design stage, and to validate and verify performance of treatment processes including as a result of changes in water quality.
- Continue to promote academic research in pathogen LRVs for drinking water treatment processes, particularly where data is limited yet LRV potential is promising. Focus should be given to soil aquifer treatment, NF, GAC. DAF and ceramic membranes as they had either smaller data sets and/or some of the widest confidence intervals.
- Standardizing the types of data that peer-reviewed journal articles of treatment technology performance report, would improve future literature reviews metadata analysis similar to this project and interpretation of results. For example, information such as source water quality parameters (e.g., temperature, turbidity, and pH), treatment technology operational parameters, number of data points collected in the study, pathogen concentrations pre- and post-treatment, and statistical methods would benefit interpretation of articles and aggregation of data. These recommended reporting standards alongside others have been summarized in a recommended checklist for authors to follow in the Supporting Information Table S6. Additionally, we recommend increased publication of raw data and inclusion of tables to present pathogen concentrations and methods of reduction calculation.

## Supplementary Material





## Notes

Copies of the search strings used to identify studies for this literature review (Table S1), the PRISMA flowchart for the literature review conducted using the search string (Figure S1), PRISMA checklist (Table S2), complete reference list of articles included in our analysis (Table S3), pathogen LRV guidance from Table 7.7 of the WHO 2022 Guidelines for Drinking Water Quality 4th edition (Table S4), bacteria and virus LRVs for bank filtration (Figure S2), LRV for pathogen types across centralized water treatment technologies based on pooled, efficacy, and effectiveness data (Table S5), recommended checklist of reporting requirements for research on water treatment technology effectiveness (Table S6) (PDF)
Complete list of publications and all extracted data included for analysis in the review (XLSX)
