# Supplementary material for: Systematic Review of Microorganism Removal Performance by Physiochemical Water Treatment Technologies
Source: Environ Sci Technol. 2025 Mar 28;59(41):21763–75. doi: 10.1021/acs.est.4c03459 (PMC12550808; doi:10.1021/acs.est.4c03459)
Supplement: Supplementary file 1 [file es4c03459_si_001.pdf]

Supporting Information for:

Systematic Review of Microorganism Removal Performance by Physiochemical Water Treatment Technologies

Matthew Burke[1], Emma Wells[1], Caleb Larison[1], Gouthami Rao[2,3], Matthew James Bentley[1], Yarrow S. Linden[1,2], Patrick Smeets[4], Jennifer De France[5], Joe Brown[2,3], Karl G. Linden[1*]

[1]University of Colorado Boulder, Boulder, CO 80303, USA

[2]University of North Carolina Chapel Hill, Chapel Hill, NC 27514, USA

[3] Georgia Institute of Technology, Atlanta, Georgia, 33032, USA

[4]KWR Water Research, Groningenhaven 7, Nieuwegein, Utrecht, NL 3430 BB Netherlands

[5]World Health Organization, Avenue Appia 20, 1211 Geneva, Switzerland

*Corresponding Author: Address: 4001 Discovery Dr., University of Colorado Boulder, Boulder, CO 80303 USA; Phone: +1 303-492-4798; karl.linden@colorado.edu

Summary: 23 pages, 2 figures, 6 tables

Pg. S2 Table S1 Copies of the search strings used to identify studies for this literature review.

Pg S3 Figure S1. PRISMA Flowchart for the literature review conducted using the search string in Table S1

Pg S4-S5 Table S2: PRISMA Checklist

Pg S6-S17 Table S3: Complete reference list of articles included in our analysis

Pg S18-S19 Table S4. Pathogen LRV guidance from Table 7.7 of the WHO's 2022 publication of the Guidelines for Drinking Water Quality.

Pg 20 Figure S2. Bacteria and Virus LRVs for Bank Filtration

Pg 21-22 Table S5. LRV for pathogen types across centralized water treatment technologies based on pooled, efficacy and effectiveness data

Pg 23 Table S6. Recommended Checklist of Reporting Requirements for Research on Water Treatment Technology Effectiveness

Table S1. Copies of the search strings used to identify studies for this literature review.

Master Search String This search string was part of a larger study and includes technologies not evaluated in this report. Another report will include the chemical disinfection technologies not addressed in this report.
TS=((water OR groundwater) AND (adsorption OR advanced oxidation OR alum OR aluminium chloride OR aluminium sulfate OR aluminium sulphate OR aluminum chloride OR aluminum sulfate OR aluminum sulphate OR anthracite OR AOP OR aquifer recharge OR aquifer storage OR BAC OR boiling OR brominated compounds OR brominated hydantoinylated resins OR bromine OR bromo-chloro compounds OR calcium hydroxide OR carbon OR chitosan OR chloramine OR chlorinated OR chlorinated hydantoinylated resins OR chlorination OR Chlorine OR chlorine dioxide OR coagulant OR coagulation OR collimated beam OR combination treatment OR copper OR diatomaceous earth OR disinfectant OR disinfection OR electrochemical OR electrolysis OR electrolytic OR electroporation OR ferrate OR ferric chloride OR ferric sulfate OR ferric sulphate OR filter OR filtration OR flocculant OR flocculation OR flotation OR forward osmosis OR heat OR hydrogen peroxide OR iodinated OR iodine compounds OR Ion Exchange OR iron OR lime OR membranes OR metals OR microfilter OR microfiltration OR microporous OR mixed media OR mixed oxidants OR mixed-oxidants OR monochloramine OR multi-barrier treatment OR multi-media OR multiple mechanisms OR nanofiltration OR oxidative catalysts OR ozonation OR ozone OR PAA OR peracetic acid OR perchlorinate OR perchlorination OR peroxide OR porous media OR power ultrasound OR reverse osmosis OR sand OR sediment infiltration OR sedimentation OR silver OR soil infiltration OR soil passage OR solar OR straining OR thermal OR titanium dioxide OR ultrafiltration OR ultraviolet OR upconversion OR UV OR UVAO OR UVAOP OR UV-LED OR zeolite) AND (Acanthamoeba OR Acinetobacter OR Adenovirus OR Aerobic spores OR Aeromonas OR Aichivirus OR Anaerobic spores OR Ascaris OR Astrovirus OR Bacteria OR Bacteriophage OR Bacteroidales OR Bacteroides OR Bacteroides fragilis OR Balantidium OR Blastocystis OR Burkholderia OR Burkholderia OR Calicivirus OR Campylobacter OR Chlamydia OR Clostridium OR Coliform OR Coliphage OR Coronavirus OR Coxsackievirus OR Cronobacter OR Cryptosporidium OR Cyanobacteria OR Cyclospora OR Cysts OR Dracunculus medinensis OR Ebola OR Echovirus OR Elizabethkingia OR Entamoeba OR Enteric virus OR Enterobacter OR Enterobacteriaceae OR Enterococci OR Enterococcus OR Enterocytozoon OR Enterovirus OR Entomeba OR Escherichia OR Faecal coliform OR Fasciola OR FC OR Fecal coliform OR Francisella OR Fungi OR Giardia OR Guinea worm OR HAV OR Helicobacter OR Hepatitis A OR Hepatitis E OR Hepevirus OR HEV OR Histoplasma OR Isospora OR Klebsiella OR Kobuvirus OR Legionella OR Leptospira OR Mamastrovirus OR Microsporidia OR Mycobacteria OR Naegleria OR Norovirus OR Norwalk virus OR Onchocerca OR Oocyst OR Orthomyxovirus OR Papovavirus OR Parechovirus OR Pasteurella OR Phage OR Polio OR Poliovirus OR Polyomavirus OR Protozoa OR Pseudomonas OR Reovirus OR Reticuloendotheliosis virus OR Rotavirus OR Salmonella OR Sapovirus OR SARS OR Schistosoma OR Shigella OR Somatic coliphage OR Spores OR Thermotolerant coliform OR Toxoplasma OR Tsukamurella OR TTC OR Vibrio OR Vibrio cholerae OR Virus OR Yersinia) AND (Small system OR Large System OR Water District OR Public Water System OR PWS OR Municipality OR Utility OR Water Utility OR Drinking Water Plant OR Potable Water Plant OR Water treatment facility OR Water treatment plant OR Water Supply OR Centralized OR Reticulated) AND (inactivation OR log kill OR lrv OR removal efficiency OR log reduction value OR percent reduction OR percent removal OR treatment effect))
Individual Technology Search String
water AND [*insert technology name*] AND (bacteria OR protozoa OR virus) AND (small-scale OR small scale OR decentralized OR point-of-consumption OR point of consumption) AND (treatment OR treatment effect OR reduction OR reduced OR log10 reduction OR log reduction OR percentage OR LRV OR log removal OR removal OR log10 removal OR disinfection or inactivation)

Figure S1. PRISMA Flowchart for the literature review conducted using the search string in Table S1

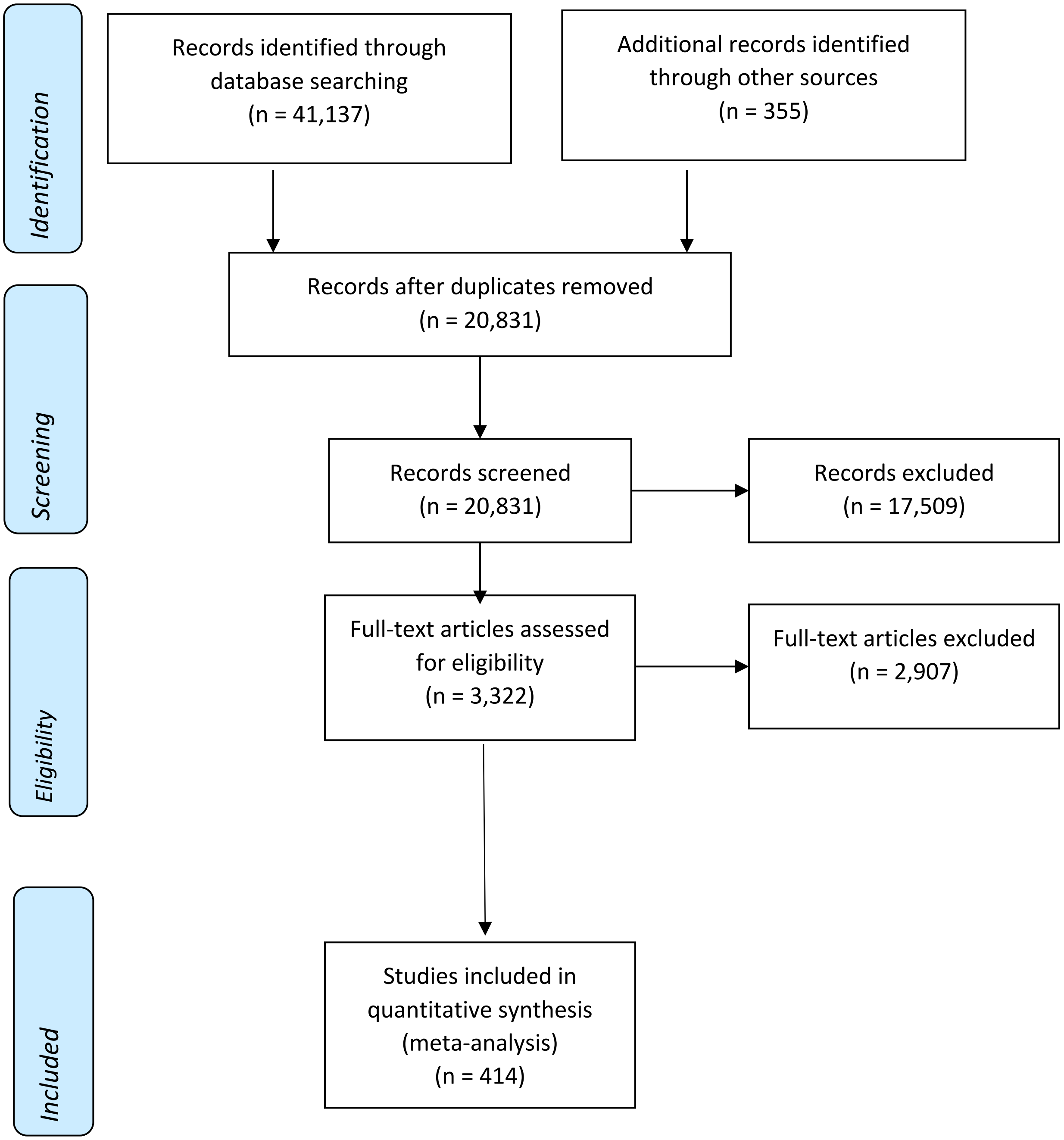

Table S2: PRISMA Checklist

Section/topic	#	Checklist item	Reported on page #
**TITLE**			
Title	1	Identify the report as a systematic review, meta-analysis, or both.	1
**ABSTRACT**			
Structured summary	2	Provide a structured summary including, as applicable: background; objectives; data sources; study eligibility criteria, participants, and interventions; study appraisal and synthesis methods; results; limitations; conclusions and implications of key findings; systematic review registration number.	
**INTRODUCTION**			
Rationale	3	Describe the rationale for the review in the context of what is already known.	2
Objectives	4	Provide an explicit statement of questions being addressed with reference to participants, interventions, comparisons, outcomes, and study design (PICOS).	2
**METHODS**			
Protocol and registration	5	Indicate if a review protocol exists, if and where it can be accessed (e.g., Web address), and, if available, provide registration information including registration number.	
Eligibility criteria	6	Specify study characteristics (e.g., PICOS, length of follow-up) and report characteristics (e.g., years considered, language, publication status) used as criteria for eligibility, giving rationale.	2-3
Information sources	7	Describe all information sources (e.g., databases with dates of coverage, contact with study authors to identify additional studies) in the search and date last searched.	2
Search	8	Present full electronic search strategy for at least one database, including any limits used, such that it could be repeated.	2
Study selection	9	State the process for selecting studies (i.e., screening, eligibility, included in systematic review, and, if applicable, included in the meta-analysis).	2
Data collection process	10	Describe method of data extraction from reports (e.g., piloted forms, independently, in duplicate) and any processes for obtaining and confirming data from investigators.	2-3
Data items	11	List and define all variables for which data were sought (e.g., PICOS, funding sources) and any assumptions and simplifications made.	2-3
Risk of bias in individual studies	12	Describe methods used for assessing risk of bias of individual studies (including specification of whether this was done at the study or outcome level), and how this information is to be used in any data synthesis.	2
Summary measures	13	State the principal summary measures (e.g., risk ratio, difference in means).	2-3

Synthesis of results	14	Describe the methods of handling data and combining results of studies, if done, including measures of consistency (e.g., I^2) for each meta-analysis.	3
Risk of bias across studies	15	Specify any assessment of risk of bias that may affect the cumulative evidence (e.g., publication bias, selective reporting within studies).	
Additional analyses	16	Describe methods of additional analyses (e.g., sensitivity or subgroup analyses, meta-regression), if done, indicating which were pre-specified.	NA
**RESULTS**			
Study selection	17	Give numbers of studies screened, assessed for eligibility, and included in the review, with reasons for exclusions at each stage, ideally with a flow diagram.	Figure S1
Study characteristics	18	For each study, present characteristics for which data were extracted (e.g., study size, PICOS, follow-up period) and provide the citations.	
Risk of bias within studies	19	Present data on risk of bias of each study and, if available, any outcome level assessment (see item 12).	NA
Results of individual studies	20	For all outcomes considered (benefits or harms), present, for each study: (a) simple summary data for each intervention group (b) effect estimates and confidence intervals, ideally with a forest plot.	
Synthesis of results	21	Present results of each meta-analysis done, including confidence intervals and measures of consistency.	15 & 17
Risk of bias across studies	22	Present results of any assessment of risk of bias across studies (see Item 15).	
Additional analysis	23	Give results of additional analyses, if done (e.g., sensitivity or subgroup analyses, meta-regression [see Item 16]).	NA
**DISCUSSION**			
Summary of evidence	24	Summarize the main findings including the strength of evidence for each main outcome; consider their relevance to key groups (e.g., healthcare providers, users, and policy makers).	8-11
Limitations	25	Discuss limitations at study and outcome level (e.g., risk of bias), and at review-level (e.g., incomplete retrieval of identified research, reporting bias).	10-11
Conclusions	26	Provide a general interpretation of the results in the context of other evidence, and implications for future research.	11
**FUNDING**			
Funding	27	Describe sources of funding for the systematic review and other support (e.g., supply of data); role of funders for the systematic review.	NA

Table S3. Complete reference list of articles included in our analysis

Roughing Filters [1–7]

Storage Reservoir[8–13]

Bank Filtration[14–16]

Clarification[17–31]

High Rate Clarification[32,33]

Dissolved Air Filtration[20,32–38]

Lime Softening[39–42]

Granular Media Filtration[17,19,25–27,34,37,43–67]

Precoat filtration[68–74]

Membrane Filtration[75–104]

Reverse Osmosis[95,105–119]

Ceramic Membrane Filtration[62,100,120–123]

Granular Activated Carbon[21,30,59,124,125]

Soil Aquifer Treatment[126–147]

Slow Sand Filtration[5,59,148–165]

(1) Jafari Dastanaie, A.; Nabi Bidhendi, G. R.; Nasrabadi, T.; Habibi, R.; Hoveidi, H. Use of Horizontal Flow Roughing Filtration in Drinking Water Treatment. *Int. J. Environ. Sci. Technol.* **2007**, *4* (3), 379–382. https://doi.org/10.1007/BF03326297.

(2) El-Taweel, G. E.; Ali, G. H. Evaluation of Roughing and Slow Sand Filters for Water Treatment. *Water, Air, & Soil Pollution* **2000**, 8. https://doi.org/10.1023/A:1005252900175.

(3) Galvis, G.; Fernandez, J.; Visscher, J. T. Comparative Study of Diffrent Pre-Treatment Alternatives. *J Water SRT - Aqua* **1993**, *42* (6), 337–346.

(4) Mushila, C. N.; Ochieng, G. M. M.; Otieno, F. A. O.; Shitote, S. M.; Sitters, C. W. Performance of Horizontal-Flow Roughing Filter Units (HRF) under Varied Raw Water Conditions in Kenya. *Journal of Global Resources* **2016**, *2*, 136–144.

(5) Ochieng, G. M. M.; Otieno, F. a. O.; Ogada, T. P. M.; Shitote, S. M.; Menzwa, D. M. Performance of Multistage Filtration Using Different Filter Media against Conventional Water Treatment Systems. *Water SA* **2004**, *30* (3), 361–367. https://doi.org/10.4314/wsa.v30i3.5085.

(6) Zeng, J.; Chen, S.; Wan, K.; Li, J.; Hu, D.; Zhang, S.; Yu, X. Study of Biological Up-Flow Roughing Filters Designed for Drinking Water Pretreatment in Rural Areas: Using Ceramic Media as Filter Material. *Environmental Technology (United Kingdom)* **2020**, *41* (10), 1256–1265. https://doi.org/10.1080/09593330.2018.1530304.

(7) Khan, Z.; Riaz, M. S.; Qaqzi, I. A. Comparing Plain and Coagulated Horizontal Flow Roughing Filtration for High Turbidity Water. *Water Science and Technology: Water Supply* **2013**, *13* (2), 413–419. https://doi.org/10.2166/ws.2013.005.

(8) Bertolucci, G. C.; Gilli, G.; Carraro, E.; Giacosa, D.; Puppo, M. Influence of Raw Water Storage on Giardia, Cryptosporidium and Nematodes. *Water Science and Technology* **1998**, *37* (2), 261–267. https://doi.org/10.2166/wst.1998.0148.

(9) Ketelaars, H. A. M.; Medema, G.; van Breemen, L. W. C. A.; van der Kooij, D.; Nobel, P. J.; Nuhn, P. Occurrence of Crypotosporidium Oocysts and Giardia Cysts in the River Meuse and Removal in the Biesbosch Reservoirs. *Journal of Water Supply: Research and Technology - AQUA* **1995**, *44* (1), 108–111.

(10) Kors, L. J.; Bosch, A. D. Catchment Protection of a Multi-Functional Reservoir. *Journal of Water Supply: Research and Technology - AQUA* **1995**, *44* (1), 80–84.

(11) Oskam, G. Main Principles of Water Quality Improvements in Reservoirs. *Journal of Water Supply: Research and Technology - AQUA* **1995**, *44* (1), 23–29.

(12) van Breemen, L. W. C. A.; Waals, J. M. J. Storage of Surface Water in the Netherlands: Challenges of the Future. *Water Supply* **1998**, *16* (1/2), 371–385.

(13) van Breemen, L.; Ketelaars, H. Storage Reservoirs – a First Barrier for Pathogenic Micro-Organisms in the Netherlands.Pdf, 1998. https://doi.org/10.1016/S0273-1223(98)00031-6.

(14) Cady, P.; Boving, T. B.; Choudri, B. S.; Cording, A.; Patil, K.; Reddy, V. Attenuation of Bacteria at a Riverbank Filtration Site in Rural India. *Water Environment Research* **2013**, *85* (11), 2164–2174.

(15) Medema; Stuyfzand. Removal of Micro-Organisms upon Basin Recharge, Deep Well Injection and River Bank Filtration in the Netherlands. In *Management of Aquifer Recharge for Sustainability*; CRC Press, 2002.

(16) Kvitsand, H. M. L.; Myrmel, M.; Fiksdal, L.; Østerhus, S. W. Evaluation of Bank Filtration as a Pretreatment Method for the Provision of Hygienically Safe Drinking Water in Norway: Results from Monitoring at Two Full-Scale Sites. *Hydrogeology Journal* **2017**, *25*, 1257–1269. https://doi.org/10.1007/s10040-017-1576-2.

(17) Asami, T.; Katayama, H.; Torrey, J. R.; Visvanathan, C.; Furumai, H. Evaluation of Virus Removal Efficiency of Coagulation-Sedimentation and Rapid Sand Filtration Processes in a Drinking Water Treatment Plant in Bangkok, Thailand. *Water Res.* **2016**, *101*, 84–94. https://doi.org/10.1016/j.watres.2016.05.012.

(18) Christensen, E.; Myrmel, M. Coagulant Residues' Influence on Virus Enumeration as Shown in a Study on Virus Removal Using Aluminium, Zirconium and Chitosan. *Journal of Water and Health* **2018**, *16* (4), 600–613. https://doi.org/10.2166/wh.2018.028.

(19) Dugan, N. R.; Fox, K. R.; Owens, J. H.; Miltner, R. J. Controlling Cryptosporidium Oocysts Using Conventional Treatment. *Journal / American Water Works Association* **2001**, *93* (12), 64–76.

(20) Edzwald, J. K.; Kelley, M. B. Control of Cryptosporidium: From Reservoirs to Clarifiers to Filters. *Water Sci. Technol.* **1998**, *37* (2), 1–8. https://doi.org/10.1016/S0273-1223(98)00004-3.

(21) Gerba, C. P.; Riley, K. R.; Nwachuku, N.; Ryu, H.; Abbaszadegan, M. Removal of Encephalitozoon Intestinalis, Calicivirus, and Coliphages by Conventional Drinking Water Treatment. *J. Environ. Sci. Health Part A-Toxic/Hazard. Subst. Environ. Eng.* **2003**, *38* (7), 1259–1268. https://doi.org/10.1081/ESE-120021124.

(22) Logsdon, G. S.; Thurman, V. C.; Frindt, E. S.; Stoecker, J. G. Evaluating Sedimentation and Various Filter Media for Removal of Giardia Cysts. *Journal - American Water Works Association* **1985**, *77* (2), 61–66. https://doi.org/10.1002/j.1551-8833.1985.tb05493.x.

(23) Mahdavi, M.; Amin, M. M.; Mahvi, A. H.; Pourzamani, H.; Ebrahimi, A. Metals, Heavy Metals and Microorganism Removal from Spent Filter Backwash Water by Hybrid Coagulation-Uf Processes. *Journal of Water Reuse and Desalination* **2018**, *8* (2), 225–233. https://doi.org/10.2166/wrd.2017.148.

(24) Medema, G. J.; Schets, F. M.; Teunis, P. F. M.; Havelaar, A. H. Sedimentation of Free and Attached Cryptosporidium Oocysts and Giardia Cysts in Water. *Appl Environ Microbiol* **1998**, *64* (11), 4460–4466.

(25) Nieminski, E. C.; Ongerth, J. E. Removing Giardia and Cryptosporidium by Conventional Treatment and Direct Filtration. *Journal - American Water Works Association* **1995**, *87* (9), 96–106.

(26) Ongerth, J. E. Evaluation of Treatment for Removing Giardia Cysts. *Journal - American Water Works Association* **1990**, *82* (6), 85–96. https://doi.org/10.1002/j.1551-8833.1990.tb06982.x.

(27) Shirasaki, N.; Matsushita, T.; Matsui, Y.; Marubayashi, T.; Murai, K. Investigation of Enteric Adenovirus and Poliovirus Removal by Coagulation Processes and Suitability of Bacteriophages MS2 and ϕX174 as Surrogates for Those Viruses. *Sci. Total Environ.* **2016**, *563–564*, 29–39. https://doi.org/10.1016/j.scitotenv.2016.04.090.

(28) Shirasaki, N.; Matsushita, T.; Matsui, Y.; Yamashita, R. Evaluation of the Suitability of a Plant Virus, Pepper Mild Mottle Virus, as a Surrogate of Human Enteric Viruses for Assessment of the Efficacy of Coagulation–Rapid Sand Filtration to Remove Those Viruses. *Water Research* **2018**, *129*, 460–469. https://doi.org/10.1016/j.watres.2017.11.043.

(29) Sylvestre, É.; Prévost, M.; Burnet, J.-B.; Pang, X.; Qiu, Y.; Smeets, P.; Medema, G.; Hachad, M.; Dorner, S. Demonstrating the Reduction of Enteric Viruses by Drinking Water Treatment during Snowmelt Episodes in Urban Areas. *Water Research X* **2021**, *11*. https://doi.org/10.1016/j.wroa.2021.100091.

(30) Abbaszadegan, M.; Monteiro, P.; Nwachuku, N.; Alum, A.; Ryu, H. Removal of Adenovirus, Calicivirus, and Bacteriophages by Conventional Drinking Water Treatment. *J. Environ. Sci. Health Part A-Toxic/Hazard. Subst. Environ. Eng.* **2008**, *43* (2), 171–177. https://doi.org/10.1080/10934520701781541.

(31) Shirasaki, N.; Matsushita, T.; Matsui, Y.; Murai, K.; Aochi, A. Elimination of Representative Contaminant Candidate List Viruses, Coxsackievirus, Echovirus, Hepatitis A Virus, and Norovirus, from Water by Coagulation Processes. *Journal of Hazardous Materials* **2017**, *326*, 110–119. https://doi.org/10.1016/j.jhazmat.2016.11.005.

(32) Edzwald, J. K.; Tobiason, J. E.; Parento, L. M.; Kelley, M. B.; Kaminski, G. S.; Dunn, H. J.; Galant, P. B. Giardia and Cryptosporidium Removals by Clarification and Filtration under Challenge Conditions. *Journal - American Water Works Association* **2000**, *92* (12), 70–84. https://doi.org/10.1002/j.1551-8833.2000.tb09072.x.

(33) Edzwald, J. K.; Tobiason, J. E.; Udden, C. T.; Kaminski, G. S.; Dunn, H. J.; Galant, P. B.; Kelley, M. B. Evaluation of the Effect of Recycle of Waste Filter Backwash Water on Plant Removals of Cryptosporidium. *Journal of Water Supply: Research and Technology-Aqua* **2003**, *52* (4), 243–258. https://doi.org/10.2166/aqua.2003.0024.

(34) Andreoli, F. C.; Sabogal-Paz, L. P. Coagulation, Flocculation, Dissolved Air Flotation and Filtration in the Removal of Giardia Spp. and Cryptosporidium Spp. from Water Supply. *Environ Technol* **2017**, 1–27. https://doi.org/10.1080/09593330.2017.1400113.

(35) Kelley, M. B.; Edzwald, J. K. The Removal Cryptosporidium by Selected Drinking Water Processes, Rensselaer Polytechnic Institute, Troy, NY, 1996.

(36) Plummer, J. D.; Edzwald, J. K.; Kelley, M. B. Removing Cryptosporidium by Dissolved-Air Flotation. *Journal - American Water Works Association* **1995**, *87* (9), 85–95. https://doi.org/10.1002/j.1551-8833.1995.tb06425.x.

(37) Hokajärvi, A.-M.; Pitkänen, T.; Meriläinen, P.; Kauppinen, A.; Matikka, V.; Kovanen, S.; Vepsäläinen, A.; Miettinen, I. T. Determination of Removal Efficiencies for Escherichia Coli, Clostridial Spores, and F-Specific Coliphages in Unit Processes of Surface Waterworks for QMRA Applications. *Water (Switzerland)* **2018**, *10* (11). https://doi.org/10.3390/w10111525.

(38) Wang, X. C.; Qiu, F. G.; Xue, X. P.; Lu, X. Application of a Virological Tracer Method for the Assessment of Pathogen Removal by Physicochemical Treatment and Chemical Disinfection. *Water Sci. Technol.* **2005**, *52* (8), 205–212.

(39) Cornwell, D. A.; MaCphee, M. J.; Brown, R. A.; Via, S. H. Demonstrating Cryptosporidium Removal Using Spore Monitoring at Lime-Softening Plants. *Journal - American Water Works Association* **2003**, *95* (5), 124–133. https://doi.org/10.1002/j.1551-8833.2003.tb10367.x.

(40) Logsdon, P. G. S.; Frey, M. M.; Stefanich, T. D.; Johnson, S. L.; Rose, J. B. The Removal and Disinfection Efficiency of Lime Softening Processes for Giardia and Viruses. **1994**, 2.

(41) Robertson, L. J.; Campbell, A. T.; Smith, H. V. Survival of Cryptosporidium Parvum Oocysts under Various Environmental Pressures. *Applied and Environmental Microbiology* **1992**, *58* (11), 3494–3500. https://doi.org/10.1128/AEM.58.11.3494-3500.1992.

(42) Sproul, O. J. Critical Review of Virus Removal by Coagulation Processes and pH Modifications, 1980.

(43) Al-Ani, M. Y.; Hendricks, D. W.; Logsdon, G. S.; Hibler, C. P. Removing Giardia Cysts From Low Turbidity Waters by Rapid Rate Filtration. *Journal - American Water Works Association* **1986**, *78* (5), 66–73. https://doi.org/10.1002/j.1551-8833.1986.tb05746.x.

(44) Amburgey, J. E. Improving Filtration for Removal of Cryptosporidium Oocysts and Particles from Drinking Water. Ph.D., Georgia Institute of Technology, United States -- Georgia. https://www.proquest.com/docview/305587514/abstract/74EFD7335F6746D6PQ/1 (accessed 2023-11-29).

(45) Arora, H.; Di Giovanni, G.; LeChevallier, M. Spent Filter Backwash Water Contaminants and Treatment Strategies. *J. Am. Water Work Assoc.* **2001**, *93* (5), 100-+.

(46) Brooks, Y. M.; Tenorio-Moncada, E. A.; Gohil, N.; Yu, Y.; Estrada-Mendez, M. R.; Bardales, G.; Richardson, R. E. Performance Evaluation of Gravity-Fed Water Treatment Systems in Rural Honduras: Verifying Robust Reduction of Turbidity and Escherichia Coli during Wet and Dry Weather. *Am J Trop Med Hyg* **2018**, *99* (4), 881–888. https://doi.org/10.4269/ajtmh.17-0577.

(47) Dugan, N. R.; Williams, D. J. Removal of Cryptosporidium by In-Line Filtration: Effects of Coagulant Type, Filter Loading Rate and Temperature. *Journal of Water Supply: Research and Technology-Aqua* **2004**, *53* (1), 1–15. https://doi.org/10.2166/aqua.2004.0001.

(48) Emelko, M. B. Removal of Viable and Inactivated Cryptosporidium by Dual- and Tri-Media Filtration. *Water Research* **2003**, *37* (12), 2998–3008. https://doi.org/10.1016/S0043-1354(03)00113-1.

(49) Gitis, V. Rapid Sand Filtration of Cryptosporidium Parvum: Effects of Media Depth and Coagulation. *Water Science and Technology: Water Supply* **2008**, *8* (2), 129–134. https://doi.org/10.2166/ws.2008.058.

(50) Gitis, V.; Adin, A.; Nasser, A.; Gun, J.; Lev, O. Fluorescent Dye Labeled Bacteriophages—a New Tracer for the Investigation of Viral Transport in Porous Media: 2. Studies of Deep-Bed Filtration. *Water Research* **2002**, *36* (17), 4235–4242. https://doi.org/10.1016/S0043-1354(02)00164-1.

(51) Assavasilavasukul, P.; Lau, B. L. T.; Harrington, G. W.; Hoffman, R. M.; Borchardt, M. A. Effect of Pathogen Concentrations on Removal of Cryptosporidium and Giardia by Conventional Drinking Water Treatment. *Water Res.* **2008**, *42* (10–11), 2678–2690. https://doi.org/10.1016/j.watres.2008.01.021.

(52) Hashimoto, A.; Hirata, T.; Kunikane, S. Occurrence of Cryptosporidium Oocysts and Giardia Cysts in a Conventional Water Purification Plant. *Water Sci. Technol.* **2001**, *43* (12), 89–92.

(53) Hendricks, D. W.; Clunie, W. F.; Sturbaum, G. D.; Klein, D. A.; Champlin, T. L.; Kugrens, P.; Hirsch, J.; McCourt, B.; Nordby, G. R.; Sobsey, M. D.; Hunt, D. J.; Allen, M. J. Filtration Removals of Microorganisms and Particles. *J. Environ. Eng.-ASCE* **2005**, *131* (12), 1621–1632. https://doi.org/10.1061/(ASCE)0733-9372(2005)131:12(1621).

(54) Hsu, B. M.; Yeh, H. H. Removal of Giardia and Cryptosporidium in Drinking Water Treatment: A Pilot-Scale Study. *Water Res.* **2003**, *37* (5), 1111–1117. https://doi.org/10.1016/S0043-1354(02)00466-9.

(55) Huck, P. M.; Coffey, B. M.; Emelko, M. B. Effects of Filter Operation on Cryptosporidium Removal. **2002**, 16.

(56) Oktaviani, A.; Suwartha, N. Analysis of Multimedia Filter Effectiveness to Improve the Quality of Rainwater Runoff in Fulfilling Urban Raw Water Supply. In *IOP Conference Series: Earth and Environmental Science*; 2021; Vol. 623. https://doi.org/10.1088/1755-1315/623/1/012008.

(57) Patania, N. L. *Optimization of Filtration for Cyst Removal*; AWWA Research Foundation and AWWA, 1995.

(58) Pineda, E.; García-Ruiz, M. J.; Guaya, D.; Manrique, J.; Osorio, F. Elimination of Total Coliforms and Escherichia Coli from Water by Means of Filtration with Natural Clays and Silica Sand in Developing Countries. *Environmental Geochemistry and Health* **2021**, *43* (1), 195–207. https://doi.org/10.1007/s10653-020-00623-1.

(59) Ramo, A.; Del Cacho, E.; Sanchez-Acedo, C.; Quilez, J. Occurrence of Cryptosporidium and Giardia in Raw and Finished Drinking Water in North-Eastern Spain. *Sci. Total Environ.* **2017**, *580*, 1007–1013. https://doi.org/10.1016/j.scitotenv.2016.12.055.

(60) Shamsollahi, H. R.; Ghoochani, M.; Sadeghi, K.; Jaafari, J.; Masinaei, M.; Sillanpää, M.; Yousefi, M.; Mirtalb, S. T.; Alimohammadi, M. Evaluation of the Physical and Chemical Characteristics of Water on the Removal Efficiency of Rotavirus in Drinking Water Treatment Plants and Change in Induced Health Risk. *Process Safety and Environmental Protection* **2019**, *130*, 6–13. https://doi.org/10.1016/j.psep.2019.07.014.

(61) Shaw, K.; Walker, S.; Koopman, B. Improving Filtration of Cryptosporidium. *Journal - American Water Works Association* **2000**, *92* (11), 103–111. https://doi.org/10.1002/j.1551-8833.2000.tb09054.x.

(62) Shirasaki, N.; Matsushita, T.; Matsui, Y.; Oshiba, A.; Ohno, K. Estimation of Norovirus Removal Performance in a Coagulation-Rapid Sand Filtration Process by Using Recombinant Norovirus VLPs. *Water Res.* **2010**, *44* (5), 1307–1316. https://doi.org/10.1016/j.watres.2009.10.038.

(63) States, S.; Tomko, R. J.; Scheuring, M.; Casson, L. Enhanced Coagulation and Removal of Cryptosporidium. *J. Am. Water Work Assoc.* **2002**, *94* (11), 67–77.

(64) Swertfeger, J.; Metz, D. H.; DeMarco, J.; Braghetta, A.; Jacangelo, J. G. Effect of Filter Media on Cyst and Oocyst Removal. **1999**, 11.

(65) Zeng, J.; Chen, Z.; Wu, S.; Ye, C.; Yu, X.; Wang, L.; Zhang, S. Removal Effect of Turbidity and Bacteria in Rural Drinking Water by GAC-Sand Filter [炭砂滤池对农村饮用水中浊度及细菌的去除效果]. *Chinese Journal of Environmental Engineering* **2020**, *14* (10), 2736–2741. https://doi.org/10.12030/j.cjee.201911049.

(66) Wang, H.; Xu, J.; Tang, W.; Li, H.; Xia, S.; Zhao, J.; Zhang, W.; Yang, Y. Removal Efficacy of Opportunistic Pathogens and Bacterial Community Dynamics in Two Drinking Water Treatment Trains. *Small* **2019**, *15* (2), e1804436. https://doi.org/10.1002/smll.201804436.

(67) Zhao, Y.; Wang, X.; Liu, C.; Wang, S.; Wang, X.; Hou, H.; Wang, J.; Li, H. Purification of Harvested Rainwater Using Slow Sand Filters with Low-Cost Materials: Bacterial Community Structure and Purifying Effect. *Science of the Total Environment* **2019**, *674*, 344–354. https://doi.org/10.1016/j.scitotenv.2019.03.474.

(68) Amburgey, J. E.; Walsh, K. J.; Fielding, R. R.; Arrowood, M. J. Removal of Cryptosporidium and Polystyrene Microspheres from Swimming Pool Water with Sand, Cartridge, and Precoat Filters. *Journal of Water and Health* **2012**, *10* (1), 31–42. https://doi.org/10.2166/wh.2011.062.

(69) DeWalle; Engeset; Lawrence. Removal of "Giardia Lambia" Cysts by Drinking Water Treatment Plants, 2002.
(70) Langé, K. P.; Bellamy, W. D.; Hendricks, D. W.; Logsdon, G. S. Diatomaceous Earth Filtration of Giardia Cysts and Other Substances. *Journal - American Water Works Association* **1986**, *78* (1), 76–84. https://doi.org/10.1002/j.1551-8833.1986.tb05682.x.
(71) Ongerth, J. E.; Hutton, P. E. Testing of Diatomaceous Earth Filtration for Removal of *Cryptosporidium* Oocysts. *Journal - American Water Works Association* **2001**, *93* (12), 54–63. https://doi.org/10.1002/j.1551-8833.2001.tb09355.x.
(72) Ongerth, J. E.; Hutton, and P. E. DE Filtration to Remove Cryptosporidium. *Journal - American Water Works Association* **1997**, *89* (12), 39–46. https://doi.org/10.1002/j.1551-8833.1997.tb08338.x.
(73) Pyper, G. Slow Sand Filter and Package Treatment Plant Evaluation: Operating Costs and Removal of Bacteria, Giardia and Trihalomethanes, 1985.
(74) Schuler, P. F.; Ghosh, iganka M. Diatomaceous Earth Filtration of Cysts and Other Particulates Using Chemical Additives. *Journal - American Water Works Association* **1990**, *82* (12), 67–75. https://doi.org/10.1002/j.1551-8833.1990.tb07069.x.
(75) Ahmed, F.; Santos, C. M.; Mangadlao, J.; Advincula, R.; Rodrigues, D. F. Antimicrobial PVK: SWNT Nanocomposite Coated Membrane for Water Purification: Performance and Toxicity Testing. *Water Research* **2013**, *47* (12), 3966–3975. https://doi.org/10.1016/j.watres.2012.10.055.
(76) Bettin, C.; Schwarz, B.; Kornmueller, A. Practical Challenge Testing of a Ceramic Membrane Module in a Full-Scale Mobile Drinking Water Treatment System. *J. Water Supply Res Technol.-Aqua* **2013**, *62* (3), 176–182. https://doi.org/10.2166/aqua.2013.126.
(77) Boudaud, N.; Machinal, C.; David, F.; Bourdonnec, A. F.-L.; Jossent, J.; Bakanga, F.; Arnal, C.; Jaffrezic, M. P.; Oberti, S.; Gantzer, C. Removal of MS2, Q Beta and GA Bacteriophages during Drinking Water Treatment at Pilot Scale. *Water Res.* **2012**, *46* (8), 2651–2664. https://doi.org/10.1016/j.watres.2012.02.020.
(78) Cruz, M. C.; Romero, L. C.; Vicente, M. S.; Rajal, V. B. Statistical Approaches to Understanding the Impact of Matrix Composition on the Disinfection of Water by Ultrafiltration. *Chemical Engineering Journal* **2017**, *316*, 305–314. https://doi.org/10.1016/j.cej.2017.01.081.
(79) Dong, X.; Al Awak, M.; Wang, P.; Sun, Y.-P.; Yang, L. Carbon Dot Incorporated Multi-Walled Carbon Nanotube Coated Filters for Bacterial Removal and Inactivation. *RSC Advances* **2018**, *8* (15), 8292–8301. https://doi.org/10.1039/c8ra00333e.
(80) ElHadidy, A. M.; Peldszus, S.; Van Dyke, M. I. Effect of Hydraulically Reversible and Hydraulically Irreversible Fouling on the Removal of MS2 and ϕX174 Bacteriophage by an Ultrafiltration Membrane. *Water Research* **2014**, *61*, 297–307. https://doi.org/10.1016/j.watres.2014.05.003.
(81) Ferrer, O.; Casas, S.; Galvañ, C.; Lucena, F.; Bosch, A.; Galofré, B.; Mesa, J.; Jofre, J.; Bernat, X. Direct Ultrafiltration Performance and Membrane Integrity Monitoring by Microbiological Analysis. *Water Res.* **2015**, *83*, 121–131. https://doi.org/10.1016/j.watres.2015.06.039.
(82) Guo, H.; Hu, J. Y. Optimization Study of a Hybrid Alum Coagulation-Membrane Filtration System for Virus Removal. *Water Sci. Technol.* **2011**, *64* (9), 1843–1850. https://doi.org/10.2166/wst.2011.147.
(83) Guo, H.; Hu, J. Effect of Hybrid Coagulation-Membrane Filtration on Downstream UV Disinfection. *Desalination* **2012**, *290*, 115–124. https://doi.org/10.1016/j.desal.2012.01.015.
(84) Guo, H.; Hu, J. Y. Fluorescent-Conjugated MS2 as Surrogates for Viruses during Drinking Water Treatment. *Water Sci. Technol.-Water Supply* **2014**, *14* (6), 991–1000. https://doi.org/10.2166/ws.2014.061.

(85) Hambsch, B.; Bösl, M.; Eberhagen, I.; Müller, U. Removal of Bacteriophages with Different Surface Charges by Diverse Ceramic Membrane Materials in Pilot Spiking Tests. *Water Science and Technology* **2012**, *66* (1), 151–157. https://doi.org/10.2166/wst.2012.141.

(86) Hirata, T.; Hashimoto, A. Experimental Assessment of the Efficacy of Microfiltration and Ultrafiltration for Cryptosporidium Removal. *Water Science and Technology* **1998**, *38* (12), 103–107.

(87) Huang, H.; Jacangelo, J. G.; Schwab, K. J. Decentralized Membrane Filtration System for Sustainable and Safe Drinking Water Supply in Low-Income Countries: Baseline Study. *J. Environ. Eng.-ASCE* **2011**, *137* (11), 981–989. https://doi.org/10.1061/(ASCE)EE.1943-7870.0000433.

(88) Jacangelo, J. G.; Aieta, E. M.; Cams, K. E.; Cummings, E. W.; Mallevialle, J. Assessing Hollow-Fiber Ultrafiltration for Particulate Removal. *Journal - American Water Works Association* **1989**, *81* (11), 68–75. https://doi.org/10.1002/j.1551-8833.1989.tb03309.x.

(89) Jacangelo, J. G.; Adham, S. S.; Laine, J.-M. Mechanism of Cryptosportidium, Giardia, and MS2 Virus Removal by MF and UF. *Journal - American Water Works Association* **1995**, *87* (9), 107–121.

(90) Karnik, B. S.; Davies, S. H.; Baumann, M. J.; Masten, S. J. Removal of Escherichia Coli after Treatment Using Ozonation-Ultrafiltration with Iron Oxide-Coated Membranes. *Ozone: Science and Engineering* **2007**, *29* (2), 75–84. https://doi.org/10.1080/01919510601139492.

(91) Kruithof, J. C.; Schippers, J. C.; Kamp, P. C.; Folmer, H. C.; Hofman, J. Integrated Multi-Objective Membrane Systems for Surface Water Treatment: Pretreatment of Reverse Osmosis by Conventional Treatment and Ultrafiltration. *Desalination* **1998**, *117* (1–3), 37–48. https://doi.org/10.1016/S0011-9164(98)00065-4.

(92) Li, L. J.; Kaymak, B.; Haas, C. N. Validation of Batch Disinfection Kinetics of Escherichia Coli Inactivation by Monochloramine in a Continuous Flow System. *Environ. Eng. Sci.* **2005**, *22* (5), 567–577. https://doi.org/10.1089/ees.2005.22.567.

(93) Lu, R.; Zhang, C.; Piatkovsky, M.; Ulbricht, M.; Herzberg, M.; Nguyen, T. H. Improvement of Virus Removal Using Ultrafiltration Membranes Modified with Grafted Zwitterionic Polymer Hydrogels. *Water Res.* **2017**, *116*, 86–94. https://doi.org/10.1016/j.watres.2017.03.023.

(94) Matsushita, T.; Shirasaki, N.; Matsui, Y.; Ohno, K. Virus Inactivation during Coagulation with Aluminum Coagulants. *Chemosphere* **2011**, *85* (4), 571–576. https://doi.org/10.1016/j.chemosphere.2011.06.083.

(95) Ohkouchi, Y.; Ase, T. Determination of Log Removal Values of Bacteria by Spiral-Wound Reverse Osmosis Modules and a Hollow Fiber Ultrafiltration Module Using Escherichia Coli and Indigenous Heterotrophic Bacteria as Indicators. *Journal of Water and Health* **2020**, *18* (6), 956–967. https://doi.org/10.2166/wh.2020.153.

(96) Pascual-Benito, M.; Emiliano, P.; Casas-Mangas, R.; Dacal-Rodriguez, C.; Gracenea, M.; Araujo, R.; Valero, F.; Garcia-Aljaro, C.; Lucena, F. Assessment of Dead-End Ultrafiltration for the Detection and Quantification of Microbial Indicators and Pathogens in the Drinking Water Treatment Processes. *International Journal of Hygiene and Environmental Health* **2020**, *230*, 113628. https://doi.org/10.1016/j.ijheh.2020.113628.

(97) Patterson, C.; Anderson, A.; Sinha, R.; Muhammad, N.; Pearson, D. Nanofiltration Membranes for Removal of Color and Pathogens in Small Public Drinking Water Sources. *J. Environ. Eng.-ASCE* **2012**, *138* (1), 48–57. https://doi.org/10.1061/(ASCE)EE.1943-7870.0000463.

(98) Podaru, C.; Manea, F.; Vlaicu, I.; Patroescu, V.; Danielescu, C.; Burtica, G. Studies Regarding Surface Water Treatment Using a Microfiltration-Ultrafiltration Pilot Plant. *Environ. Eng. Manag. J.* **2008**, *7* (6), 711–715.

(99) Shirasaki, N.; Matsushita, T.; Matsui, Y.; Ohno, K.; Kobuke, M. Virus Removal in a Hybrid Coagulation-Microfiltration System - Investigating Mechanisms of Virus Removal by a Combination

of PCR and PFU Methods. *Water Science and Technology: Water Supply* **2007**, *7* (5–6), 1–8. https://doi.org/10.2166/ws.2007.136.

(100) Shirasaki, N.; Matsushita, I.; Matsui, Y.; Kobuke, M.; Ohno, K. Comparison of Removal Performance of Two Surrogates for Pathogenic Waterborne Viruses, Bacteriophage Q Beta and MS2, in a Coagulation-Ceramic Microfiltration System. *J. Membr. Sci.* **2009**, *326* (2), 564–571. https://doi.org/10.1016/j.memsci.2008.10.037.

(101) Shirasaki, N.; Matsushita, T.; Matsui, Y.; Urasaki, T.; Ohno, K. Difference in Behaviors of F-Specific DNA and RNA Bacteriophages during Coagulation-Rapid Sand Filtration and Coagulation-Microfiltration Processes. *Water Science and Technology: Water Supply* **2012**, *12* (5), 666–673. https://doi.org/10.2166/ws.2012.041.

(102) Tanneru, C. T.; Rimer, J. D.; Chellam, S. Sweep Flocculation and Adsorption of Viruses on Aluminum Flocs during Electrochemical Treatment Prior to Surface Water Microfiltration. *Environmental Science and Technology* **2013**, *47* (9), 4612–4618. https://doi.org/10.1021/es400291e.

(103) Yin, Z.; Tarabara, V. V.; Xagoraraki, I. Human Adenovirus Removal by Hollow Fiber Membranes: Effect of Membrane Fouling by Suspended and Dissolved Matter. *J. Membr. Sci.* **2015**, *482*, 120–127. https://doi.org/10.1016/j.memsci.2015.02.028.

(104) Zhu, B.; Clifford, D. A.; Chellam, S. Comparison of Electrocoagulation and Chemical Coagulation Pretreatment for Enhanced Virus Removal Using Microfiltration Membranes. *Water Research* **2005**, *39* (13), 3098–3108. https://doi.org/10.1016/j.watres.2005.05.020.

(105) Adham, S. S.; Gagliardo, G.; Smith, D.; Ross, D.; Gramith, K.; Trussell, R. Monitoring the Integrity of Reverse Osmosis Membranes. *Desalination* **1998**, *119* (1–3), 143–150. https://doi.org/10.1016/S0011-9164(98)00134-9.

(106) Adham, S.; Trussell, S.; Gagliardo, P.; Trussell, R. Rejection of MS-2 Virus by RO Membranes. *Journal AWWA* **1998**, *90* (9), 130–135.

(107) Antony, A.; Branch, A.; Leslie, G.; Le-Clech, P. Impact of Membrane Ageing on Reverse Osmosis Performance – Implications on Validation Protocol. *Journal of Membrane Science* **2016**, *520*, 37–44. https://doi.org/10.1016/j.memsci.2016.07.036.

(108) Casani, S.; Hansen, T. B.; Christensen, J.; Knøchel, S. Comparison of Methods for Assessing Reverse Osmosis Membrane Treatment of Shrimp Process Water. *J Food Prot* **2005**, *68* (4), 801–807. https://doi.org/10.4315/0362-028x-68.4.801.

(109) Comerton, A. M.; Andrews, R. C.; Bagley, D. M. Evaluation of an MBR-RO System to Produce High Quality Reuse Water: Microbial Control, DBP Formation and Nitrate. *Water Res* **2005**, *39* (16), 3982–3990. https://doi.org/10.1016/j.watres.2005.07.014.

(110) Fujioka, T.; Makabe, R.; Mori, N.; Snyder, S. A.; Leddy, M. Assessment of Online Bacterial Particle Counts for Monitoring the Performance of Reverse Osmosis Membrane Process in Potable Reuse. *Sci Total Environ* **2019**, *667*, 540–544. https://doi.org/10.1016/j.scitotenv.2019.02.339.

(111) Governal, R. A.; Gerba, C. P. Removal of MS-2 and PRD-1 Bacteriophages from an Ultrapure Water System. *J Ind Microbiol Biotech* **1999**, *23* (3), 166–172. https://doi.org/10.1038/sj.jim.2900711.

(112) Hörman, A.; Nevas, M.; Lindström, M.; Hänninen, M.-L.; Korkeala, H. Elimination of Botulinum Neurotoxin (BoNT) Type B from Drinking Water by Small-Scale (Personal-Use) Water Purification Devices and Detection of BoNT in Water Samples. *Appl. Environ. Microbiol.* **2005**, *71* (4), 1941–1945. https://doi.org/10.1128/AEM.71.4.1941-1945.2005.

(113) Hornstra, L. M.; da Silva, T. R.; Blankert, B.; Heijnen, L.; Beerendonk, E.; Cornelissen, E. R.; Medema, G. Monitoring the Integrity of Reverse Osmosis Membranes Using Novel Indigenous Freshwater Viruses and Bacteriophages. *Environmental Science-Water Research & Technology* **2019**, *5* (9), 1535–1544. https://doi.org/10.1039/c9ew00318e.

(114) Iranpour, R. Virus Removal by Advanced Membrane Filtration for Wastewater Reclamation. *Water Environment Research* **1998**, *70* (6), 1198–1204.

(115) Kitis, M.; Lozier, J. C.; Kim, J.-H.; Mi, B.; Mariñas, B. J. Microbial Removal and Integrity Monitoring of Ro and NF Membranes. *Journal AWWA* **2003**, *95* (12), 105–119. https://doi.org/10.1002/j.1551-8833.2003.tb10515.x.
(116) März, F.; Scheer, R.; Graf, E. Use of a Laser Diffraction Particle Counter to Monitor Integrity of Reverse Osmosis Membranes and Its Inefficiency to Detect Bacteria in Product Water. *International Journal of Pharmaceutics* **1990**, *61* (1), 57–66. https://doi.org/10.1016/0378-5173(90)90044-5.
(117) Mi, B.; Eaton, C. L.; Kim, J.-H.; Colvin, C. K.; Lozier, J. C.; Mariñas, B. J. Removal of Biological and Non-Biological Viral Surrogates by Spiral-Wound Reverse Osmosis Membrane Elements with Intact and Compromised Integrity. *Water Res* **2004**, *38* (18), 3821–3832. https://doi.org/10.1016/j.watres.2004.07.004.
(118) Pype, M.-L.; Donose, B. C.; Martí, L.; Patureau, D.; Wery, N.; Gernjak, W. Virus Removal and Integrity in Aged RO Membranes. *Water Research* **2016**, *90*, 167–175. https://doi.org/10.1016/j.watres.2015.12.023.
(119) Sorber, C. A.; Malina, J. F.; Sagik, B. P. Virus Rejection by the Reverse Osmosis-Ultrafiltration Processes. *Water Research* **1972**, *6* (11), 1377–1388. https://doi.org/10.1016/0043-1354(72)90200-X.
(120) Bartels, J.; Batista, A. G.; Kroll, S.; Maas, M.; Rezwan, K. Hydrophobic Ceramic Capillary Membranes for Versatile Virus Filtration. *Journal of Membrane Science* **2019**, *570–571*, 85–92. https://doi.org/10.1016/j.memsci.2018.10.022.
(121) Matsushita, T.; Matsui, Y.; Shirasaki, N.; Kato, Y. Effect of Membrane Pore Size, Coagulation Time, and Coagulant Dose on Virus Removal by a Coagulation-Ceramic Microfiltration Hybrid System. *Desalination* **2005**, *178* (1–3), 21–26. https://doi.org/10.1016/j.desal.2004.11.026.
(122) Meyn, T.; Leiknes, T. O.; Koenig, A. MS2 Removal from High NOM Content Surface Water by Coagulation - Ceramic Microfiltration, for Potable Water Production. *AICHE J.* **2012**, *58* (7), 2270–2281. https://doi.org/10.1002/aic.12731.
(123) Werner, J.; Besser, B.; Brandes, C.; Kroll, S.; Rezwan, K. Production of Ceramic Membranes with Different Pore Sizes for Virus Retention. *Journal of Water Process Engineering* **2014**, *4* (C), 201–211. https://doi.org/10.1016/j.jwpe.2014.10.007.
(124) El-Zanfaly, H. T.; Reasoner, D. J.; Geldreich, E. E. Bacteriological Changes Associated with Granular Activated Carbon in a Pilot Water Treatment Plant. *Water Air Soil Pollut.* **1998**, *107* (1–4), 73–80.
(125) Hijnen, W. a. M.; Suylen, G. M. H.; Bahlman, J. A.; Brouwer-Hanzens, A.; Medema, G. J. GAC Adsorption Filters as Barriers for Viruses, Bacteria and Protozoan (Oo)Cysts in Water Treatment. *Water Res.* **2010**, *44* (4), 1224–1234. https://doi.org/10.1016/j.watres.2009.10.011.
(126) Auckenthaler, A.; Raso, G.; Huggenberger, P. Particle Transport in a Karst Aquifer: Natural and Artificial Tracer Experiments with Bacteria, Bacteriophages and Microspheres. *Water Sci Technol* **2002**, *46* (3), 131–138.
(127) Bales, R. C.; Li, S.; Maguire, K. M.; Yahya, M. T.; Gerba, C. P.; Harvey, R. W. Virus and Bacteria Transport in a Sandy Aquifer, Cape Cod, MA. *Groundwater* **1995**, *33* (4), 653–661. https://doi.org/10.1111/j.1745-6584.1995.tb00321.x.
(128) Bales, R. C.; Li, S.; Yeh, T.-C. J.; Lenczewski, M. E.; Gerba, C. P. Bacteriophage and Microsphere Transport in Saturated Porous Media: Forced-Gradient Experiment at Borden, Ontario. *Water Resources Research* **1997**, *33* (4), 639–648. https://doi.org/10.1029/97WR00025.
(129) Champ, D. R.; Schroeter, J. Bacterial Transport in Fractured Rock – A Field-Scale Tracer Test at the Chalk River Nuclear Laboratories. *Water Science and Technology* **1988**, *20* (11–12), 81–87. https://doi.org/10.2166/wst.1988.0269.

(130) DeBorde, D. C.; Woessner, W. W.; Kiley, Q. T.; Ball, P. Rapid Transport of Viruses in a Floodplain Aquifer. *Water Research* **1999**, *33* (10), 2229–2238. https://doi.org/10.1016/S0043-1354(98)00450-3.

(131) Harvey, R. W.; Kinner, N. E.; MacDonald, D.; Metge, D. W.; Bunn, A. Role of Physical Heterogeneity in the Interpretation of Small-Scale Laboratory and Field Observations of Bacteria, Microbial-Sized Microsphere, and Bromide Transport through Aquifer Sediments. *Water Resources Research* **1993**, *29* (8), 2713–2721. https://doi.org/10.1029/93WR00963.

(132) Hornstra, L. M.; Schijven, J. F.; Waade, A.; Prat, G. S.; Smits, F. J. C.; Cirkel, G.; Stuyfzand, P. J.; Medema, G. J. Transport of Bacteriophage MS2 and PRD1 in Saturated Dune Sand under Suboxic Conditions. *Water Research* **2018**, *139*, 158–167. https://doi.org/10.1016/j.watres.2018.03.054.

(133) Mallén, G.; Maloszewski, P.; Flynn, R.; Rossi, P.; Engel, M.; Seiler, K.-P. Determination of Bacterial and Viral Transport Parameters in a Gravel Aquifer Assuming Linear Kinetic Sorption and Desorption. *Journal of Hydrology* **2005**, *306* (1), 21–36. https://doi.org/10.1016/j.jhydrol.2004.08.033.

(134) McKay, L. D.; Cherry, J. A.; Bales, R. C.; Yahya, M. T.; Gerba, C. P. A Field Example of Bacteriophage as Tracers of Fracture Flow. *Environ. Sci. Technol.* **1993**, *27* (6), 1075–1079. https://doi.org/10.1021/es00043a006.

(135) McKay, L. d.; Sanford, W. e.; Strong, J. m. Field-Scale Migration of Colloidal Tracers in a Fractured Shale Saprolite. *Groundwater* **2000**, *38* (1), 139–147. https://doi.org/10.1111/j.1745-6584.2000.tb00211.x.

(136) Pang. Protection Zones of the Major Water Supply Springs in the Rotorua District. *Institute of Environmental Science and Research 96* (7).

(137) Schijven, J. F.; Hoogenboezem, W.; Nobel, P. J.; Medema, G. J.; Stakelbeek, A. Reduction of FRNA-Bacteriophages and Faecal Indicator Bacteria by Dune Infiltration and Estimation of Sticking Efficiencies. *Water Science and Technology* **1998**, *38* (12), 127–131. https://doi.org/10.1016/S0273-1223(98)00813-0.

(138) Schijven, J. F.; Hoogenboezem, W.; Hassanizadeh, M.; Peters, J. H. Modeling Removal of Bacteriophages MS2 and PRD1 by Dune Recharge at Castricum, Netherlands. *Water Resources Research* **1999**, *35* (4), 1101–1111. https://doi.org/10.1029/1998WR900108.

(139) Schijven, J. F.; Medema, G.; Vogelaar, A. J.; Hassanizadeh, S. M. Removal of Microorganisms by Deep Well Injection. *Journal of Contaminant Hydrology* **2000**, *44* (3), 301–327. https://doi.org/10.1016/S0169-7722(00)00098-X.

(140) Sinton, L. W. *Investigations into the Use of the Bacterial Species Bacillus Stearothermophilus and Escherichia Coli (H2S Positive) as Tracers of Groundwater Movement*; Water & soil technical publication; published for the National Water and Soil Conservation Organisation by the Water and Soil Division, Ministry of Works and Development: Wellington, N.Z, 1980.

(141) Sinton, L. W.; Close, M. E. Groundwater Tracing Experiments. *Publication ... of the Hydrology Centre, Christchurch* **1983**.

(142) Sinton, L. W.; Noonan, M. J.; Finlay, R. K.; Pang, L.; Close, M. E. Transport and Attenuation of Bacteria and Bacteriophages in an Alluvial Gravel Aquifer. *New Zealand Journal of Marine and Freshwater Research* **2000**, *34* (1), 175–186. https://doi.org/10.1080/00288330.2000.9516924.

(143) Van der Wielen, P. W. J. J.; Senden, W. J. M. K.; Medema, G. Removal of Bacteriophages MS2 and ΦX174 during Transport in a Sandy Anoxic Aquifer. *Environ. Sci. Technol.* **2008**, *42* (12), 4589–4594. https://doi.org/10.1021/es800156c.

(144) Wall, K.; Pang, L.; Sinton, L.; Close, M. Transport and Attenuation of Microbial Tracers and Effluent Microorganisms in Saturated Pumice Sand Aquifer Material. *Water Air Soil Pollut* **2008**, *188* (1), 213–224. https://doi.org/10.1007/s11270-007-9537-3.

(145) Woessner, W. W.; Ball, P. N.; DeBorde, D. C.; Troy, T. L. Viral Transport in a Sand and Gravel Aquifer under Field Pumping Conditions. *Ground Water* **2001**, *39* (6), 886–894. https://doi.org/10.1111/j.1745-6584.2001.tb02476.x.
(146) Zhang, P.; Johnson, W. P.; Scheibe, T. D.; Choi, K.-H.; Dobbs, F. C.; Mailloux, B. J. Extended Tailing of Bacteria Following Breakthrough at the Narrow Channel Focus Area, Oyster, Virginia. *Water Resources Research* **2001**, *37* (11), 2687–2698. https://doi.org/10.1029/2000WR000151.
(147) Flynn, R. Virus Transport and Attenuation in Perialpine Gravel Aquifers. **2003**.
(148) Heller, L.; Cardoso Martins Vieira, M. B.; Alves de Brito, L. L.; Salvador, D. P. Association between the Concentration of Protozoa and Surrogates in Effluents of the Slow Sand Filtration for Water Treatment. *Braz. J. Microbiol.* **2007**, *38* (2), 337–345. https://doi.org/10.1590/S1517-83822007000200029.
(149) Dizer, H.; Schmidt, R.; Szewzyk, R.; López-Pila, J. M. Comparison of Microbial Removal between Slow Dead-End versus Tangential Sand Filtration. *Water Environment Research* **2018**, *90* (7), 589–597. https://doi.org/10.2175/106143017X15131012153086.
(150) Seelaus, T. J.; Hendricks, D. W.; Janonis, B. A. Design and Operation of a Slow Sand Filter. *Journal - American Water Works Association* **1986**, *78* (12), 35–41. https://doi.org/10.1002/j.1551-8833.1986.tb02763.x.
(151) Hijnen, W. A. M.; Schijven, J. F.; Bonné, P.; Visser, A.; Medema, G. J. Elimination of Viruses, Bacteria and Protozoan Oocysts by Slow Sand Filtration. *Water Science and Technology* **2004**, *50* (1), 147–154.
(152) Tanner, S. A.; Ongerth, J. E. Evaluating the Performance of Slow Sand Filters in Northern Idaho. *Journal - American Water Works Association* **1990**, *82* (12), 51–61. https://doi.org/10.1002/j.1551-8833.1990.tb07067.x.
(153) Anderson, W. B.; Deloyde, J. L.; Van Dyke, M. I.; Huck, P. M. Influence of Design and Operating Conditions on the Removal of MS2 Bacteriophage by Pilot-Scale Multistage Slow Sand Filtration. *Journal of Water Supply: Research and Technology - AQUA* **2009**, *58* (7), 450–462. https://doi.org/10.2166/aqua.2009.140.
(154) Chan, S.; Pullerits, K.; Riechelmann, J.; Persson, K. M.; Rådström, P.; Paul, C. J. Monitoring Biofilm Function in New and Matured Full-Scale Slow Sand Filters Using Flow Cytometric Histogram Image Comparison (CHIC). *Water Research* **2018**, *138*, 27–36. https://doi.org/10.1016/j.watres.2018.03.032.
(155) D'Alessio, M.; Yoneyama, B.; Kirs, M.; Kisand, V.; Ray, C. Pharmaceutically Active Compounds: Their Removal during Slow Sand Filtration and Their Impact on Slow Sand Filtration Bacterial Removal. *Sci. Total Environ.* **2015**, *524–525*, 124–135. https://doi.org/10.1016/j.scitotenv.2015.04.014.
(156) Hijnen, W. A. M.; Dullemont, Y. J.; Schijven, J. F.; Hanzens-Brower, A. J.; Rosielle, M.; Medema, G. Removal and Fate of Cryptosporidium Paruum, Clostridium Perfringens and Small-Sized Centric Diatoms (Stephanodiscus Hantzschii) in Slow Sand Filters. *Water Res.* **2007**, *41* (10), 2151–2162. https://doi.org/10.1016/j.watres.2007.01.056.
(157) Bauer, R.; Dizer, H.; Graeber, I.; Rosenwinkel, K.-H.; Lopez-Pila, J. M. Removal of Bacterial Fecal Indicators, Coliphages and Enteric Adenoviruses from Waters with High Fecal Pollution by Slow Sand Filtration. *Water Res.* **2011**, *45* (2), 439–452. https://doi.org/10.1016/j.watres.2010.08.047.
(158) Pereira, S. P.; Martins, F. de C.; Ludolf Gomes, L. N.; Sales, M. do V.; De Padua, V. L. Removal of Cyanobacteria by Slow Sand Filtration for Drinking Water. *J. Wate Sanit. Hyg. Dev.* **2012**, *2* (3), 133–145. https://doi.org/10.2166/washdev.2012.047.
(159) Fogel, D.; Isaac-Renton, J.; Guasparini, R.; Moorehead, W.; Ongerth, J. Removing Giardia and Cryptosporidium by Slow Sand Filtration. *Journal - American Water Works Association* **1993**, *85* (11), 77–84. https://doi.org/10.1002/j.1551-8833.1993.tb06105.x.

(160) Bellamy, W. D.; Silverman, G. P.; Hendricks, D. W.; Logsdon, G. S. Removing Giardia Cysts With Slow Sand Filtration. *Journal - American Water Works Association* **1985**, *77* (2), 52–60. https://doi.org/10.1002/j.1551-8833.1985.tb05492.x.
(161) Cleasby, J. L.; Hilmoe, D. J.; Dimitracopoulos, C. J. Slow Sand and Direct In-Line Filtration of a Surface Water. *Journal - American Water Works Association* **1984**, *76* (12), 44–55. https://doi.org/10.1002/j.1551-8833.1984.tb05455.x.
(162) Ohlenschlger, M.; Christensen, S. C. B.; Bregnhoj, H.; Albrechtsen, H.-J. Submerged Pond Sand Filter-A Novel Approach to Rural Water Supply. *Water* **2016**, *8* (6), 250. https://doi.org/10.3390/w8060250.
(163) Heller, L.; de Brito, L. L. A. The Retention of Cryptosporidium Sp Oocysts at Varying Depths in Slow Sand Filters: A Pilot Study. *J. Water Supply Res Technol.-Aqua* **2006**, *55* (3), 193–206. https://doi.org/10.2166/aqua.2006.070.
(164) Urfer, D. Use of Bauxite for Enhanced Removal of Bacteria in Slow Sand Filters. *Water Science and Technology: Water Supply* **2017**, *17* (4), 1007–1015. https://doi.org/10.2166/ws.2016.199.
(165) Canh, V. D.; Furumai, H.; Katayama, H. Removal of Pepper Mild Mottle Virus by Full-Scale Microfiltration and Slow Sand Filtration Plants. *npj Clean Water* **2019**, *2* (1). https://doi.org/10.1038/s41545-019-0042-1.

Table S4. Pathogen LRV guidance from Table 7.7 of the WHO's 2022 publication of the Guidelines for Drinking Water Quality.

Table 7.7 Reductions of bacteria, viruses and protozoa achieved by water treatment technologies at drinking-water treatment plants for large communities

Treatment process	Enteric pathogen group	Minimum removal (LRV)	Maximum removal (LRV)	Notes
**Pretreatment**				
Roughing filters	Bacteria	0.2	2.3	Depends on filter medium, coagulant
Storage reservoirs	Bacteria	0.7	2.2	Residence time > 40 days
	Protozoa	1.4	2.3	Residence time 160 days
Bank filtration	Viruses	> 2.1	8.3	Depends on travel distance, soil type, pumping rate, pH, ionic strength
	Bacteria	2	> 6	
	Protozoa	> 1	> 2	
**Coagulation, flocculation and sedimentation**				
Conventional clarification	Viruses	0.1	3.4	Depends on coagulation conditions
	Bacteria	0.2	2	
	Protozoa	1	2	
High-rate clarification	Protozoa	> 2	2.8	Depends on use of appropriate blanket polymer
Dissolved air flotation	Protozoa	0.6	2.6	Depends on coagulant dose
Lime softening	Viruses	2	4	Depends on pH and settling time
	Bacteria	1	4	
	Protozoa	0	2	

Table 7.7 (continued)

Treatment process	Enteric pathogen group	Minimum removal (LRV)	Maximum removal (LRV)	Notes
**Filtration**				
Granular high-rate filtration	Viruses	0	3.5	Depends on filter media and coagulation pretreatment; filtered water turbidity of ≤ 0.3 NTU in 95% of samples (and none to exceed 1 NTU) associated with 1–2 log reduction of viruses and 3 log reduction of *Cryptosporidium*[a]
	Bacteria	0.2	4.4	
	Protozoa	0.4	3.3	
Slow sand filtration	Viruses	0.25	4	Depends on presence of schmutzdecke, grain size, flow rate, operating conditions (mainly temperature, pH); filtered water turbidity of ≤ 1NTU in 95% of samples (and none to exceed 5 NTU) associated with 1–2 log reduction of viruses and 2.5–3 log reduction of *Cryptosporidium*[a]
	Bacteria	2	6	
	Protozoa	0.3	> 5	
Precoat filtration	Viruses	1	1.7	If filter cake is present
	Bacteria	0.2	2.3	Depends on chemical pretreatment
	Protozoa	3	6.7	Depends on media grade and filtration rate
Membrane filtration: microfiltration, ultrafiltration, nanofiltration, reverse osmosis	Viruses	< 1	> 6.5	Varies with membrane pore size (microfilters, ultrafilters, nanofilters and reverse osmosis filters), integrity of filter medium and filter seals, and resistance to chemical and biological ("grow-through") degradation; maximum reductions associated with filtered water turbidity of < 0.1 NTU[a]
	Bacteria	1	> 7	
	Protozoa	2.3	> 7	

Figure S2. Bacteria and Virus LRVs for Bank Filtration

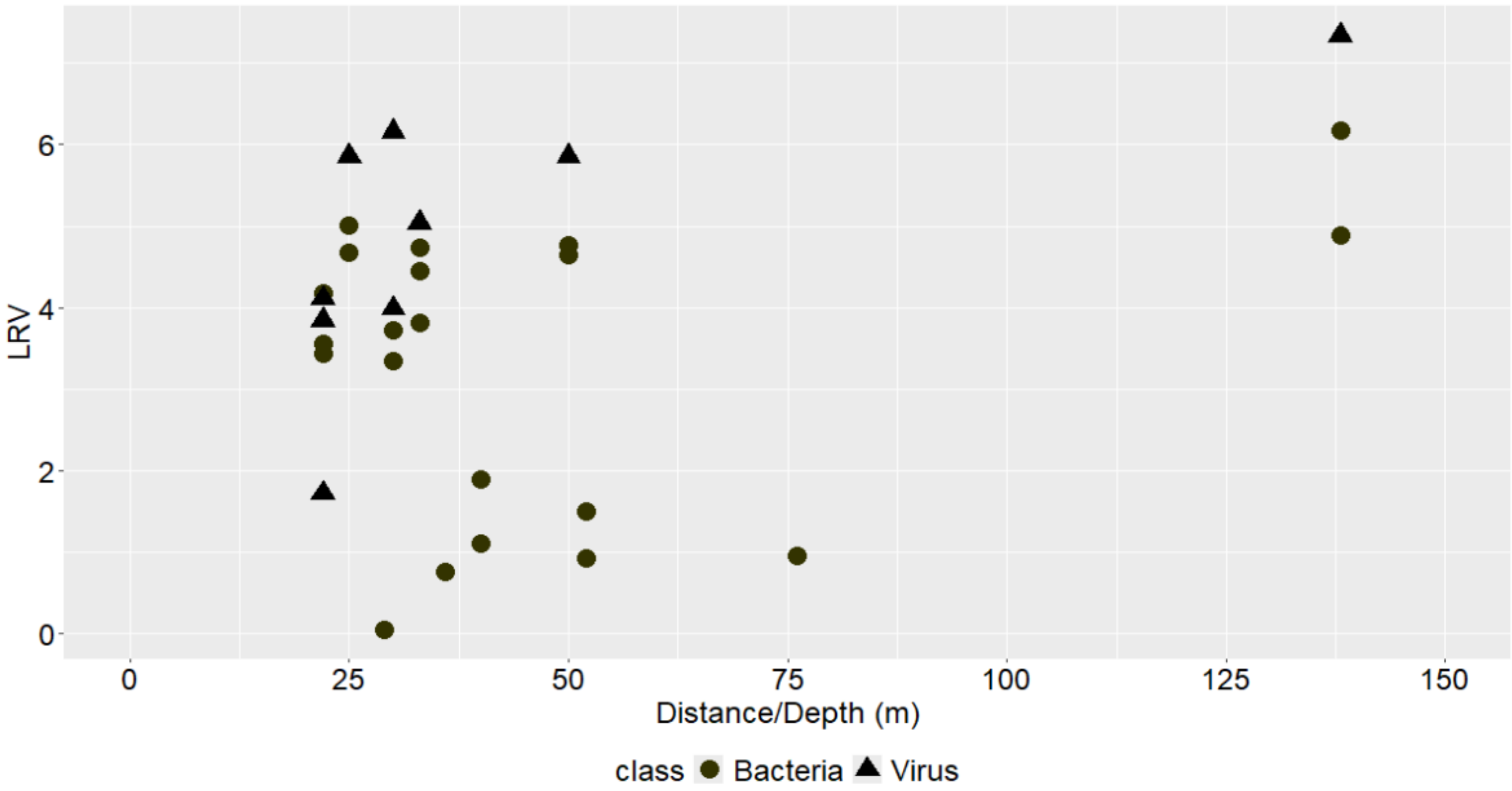

Table S5. LRV for pathogen types across centralized water treatment technologies based on pooled, efficacy and effectiveness data

Technology	Pathogen group	Total number of studies	Total number of point estimates	Mean $\log_{10}$ reduction value: all studies (95% CI)	n	Mean $\log_{10}$ reduction value: controlled laboratory conditions (95% CI)	n	Mean $\log_{10}$ reduction value: field conditions (95% CI)
Roughing Filter	Bacteria	7	53	0.9 (0.8-1.0)			53	0.9 (0.8-1.0)
Storage Reservoirs	Bacteria	4	19	1.5 (1.0-2.0)	-	-	19	1.5 (1.0-2.0)
	Protozoa	4	12	1.7 (1.2-2.1)	-	-	12	1.7 (1.2-2.1)
	Virus	2	2	2.3*	-	-	2	2.3*
Bank Filtration	Bacteria	3	15	1.7 (1.2-2.2)	-	-	15	1.7 (1.2-2.2)
	Virus	2	8	2.9*	-	-	8	2.9*
Conventional Clarification	Bacteria	3	10	1.1 (0.8-1.4)	-	-	10	1.1 (0.8-1.4)
	Protozoa	7	44	1.1 (0.8-1.3)	8	0.8 (0.2-1.3)	36	1.1 (0.8-1.4)
	Virus	6	243	1.6 (1.5-1.7)	84	0.9 (0.8-1.1)	159	1.9 (1.8-2.1)
High-rate Clarification	Protozoa	2	12	1.2 (0.9-1.4)	-	-	12	1.2 (0.9-1.4)
Dissolved Air Flotation	Bacteria	1	3	2.7*	-	-	3	2.7*
	Protozoa	7	60	2.4 (2.2-2.6)	48	2.5 (2.2-2.7)	12	2.2 (2.0-2.4)
	Virus	2	4	2.5*	1	1.8*	3	2.8*
Lime Softening	Bacteria	1	15	2.6 (1.9-3.3)			15	2.6 (1.9-3.3)
	Protozoa	3	12	1.1(0.5-1.6)	1	0.3*	11	1.1 (0.6-1.7)
	Virus	1	5	2*	5	2.0*	-	-
Granular Media	Bacteria	6	61	1.8 (1.5-2.1)	34	1.6 (1.2-1.9)	27	2.1 (1.7-2.5)
	Protozoa	19	146	3 (2.8-3.3)	42	2.0 (1.5-2.4)	104	3.4 (3.1-3.8)
	Virus	5	45	2.6 (2.3-3.0)	37	2.8 (2.4-3.1)	8	2.1*
Slow Sand Filtration	Bacteria	15	132	1.7 (1.6-1.9)	26	2.4 (1.8-3.0)	106	1.6 (1.4-1.8)
	Protozoa	7	65	2.6 (2.4-2.9)	54	2.9 (2.7-3.0)	11	1.5 (0.6-2.4)
	Virus	4	25	2 (1.4-2.5)	8	3.4*	17	1.3 (0.9-1.7)
Precoat Filtration	Bacteria	3	73	1.3 (1.2-1.5)	28	1.5(1.2-1.8)	45	1.2 (1.1-1.4)
	Protozoa	7	82	4.4 (4.1-4.7)	81	4.4(4.1-4.7)	1	3.5*
Membrane filtration (MF, UF, NF, RO combined)	Bacteria	25	58	4.5 (3.9-5.1)	46	4.7 (4.0-5.4)	12	3.6 (2.3-4.9)
	Protozoa	5	43	5.7 (5.4-6.0)	43	5.7 (5.4-6.0)	-	-

	Virus	52	167	2.8 (2.5-3.1)	92	2.9 (2.5-3.3)	75	2.6 (2.1-3.0)
Microfiltration (MF)	Bacteria	1	7	4.7*	7	4.7*	-	-
	Protozoa	1	24	5.7 (5.3-6.1)	24	5.7 (5.3-6.1)	-	-
	Virus	9	44	1.8 (1.3-2.3)	42	1.9 (1.3-2.4)	2	0.2
Ultrafiltration (UF)	Bacteria	11	36	4.6 (3.7-5.5)	28	4.7 (3.7-5.8)	8	4.1 (2.6-5.5)
	Protozoa	2	19	5.8 (5.3-6.2)	19	5.8 (5.3-6.2)	-	-
	Virus	17	107	2.8 (2.5-3.2)	43	3.6 (3.1-4.1)	64	2.4 (1.9-2.8)
Nanofiltration (NF)	Bacteria	1	3	3.1*	3	3.1	-	-
	Virus	1	1	4.6*	1	4.6	-	-
Reverse Osmosis (RO)	Bacteria	5	11	4.8 (3.5-6.1)	10	5.2 (4.1-6.3)	1	1.2
	Protozoa	1	1	5.7			1	5.7
	Virus	11	15	4.9 (4.0-5.7)	7	5.1*	8	4.7*
Granular Activated Carbon	Bacteria	3	11	0.6 (0.2-1.1)	-	-	11	0.6 (0.2-1.1)
	Protozoa	2	10	1.5 (0.9-2.1)	-	-	10	1.5 (0.9-2.1)
	Virus	2	20	3.1 (2.4-3.8)	-	-	20	3.1 (2.4-3.8)
Ceramic Membrane	Virus	6	50	4.7 (4.1-5.3)	46	4.7 (4.1-5.4)	4	4.2*
Soil Aquifer Treatment	Bacteria	13	21	2.4 (1.5-3.4)	-	-	21	2.4 (1.5-3.4)
	Protozoa	1	1	0.4	-	-	1	0.4
	Virus	18	92	4.3 (3.8-4.8)	-	-	92	4.3 (3.8-4.8)

*Note less than 10 data points – therefore the mean LRV values presented may be skewed by outliers and no 95% CI is presented

Table S6. Recommended Checklist of Reporting Requirements for Research on Water Treatment Technology Effectiveness

METHODS

- ☐ Challenge water description
- ☐ Water chemistry parameters defined and methods specified
- ☐ Specific and technically accurate description of treatment technology, including known or suspected mechanism
- ☐ Dose or CT and units
- ☐ Target microbe; source; state (e.g., vegetative, spore); how isolated and handled and grown
- ☐ Microbe in biofilm/aggregate or dispersed
- ☐ Application/testing context
- ☐ Scale
- ☐ Microbial assay type and reference, with appropriate methodological details
- ☐ Number matched pre-post treatment pairs per unit
- ☐ Number units reported
- ☐ Analytical volume
- ☐ LRV and indication of whether it is log10 or ln
- ☐ Time over LRV demonstrated
- ☐ Volume over LRV demonstrated
- ☐ Positive controls reported
- ☐ Negative controls reported
- ☐ Limit of quantification/Limit of detection defined and reported
- ☐ Specify geometric or arithmetic mean used in the analysis

RESULTS

- ☐ Includes standard deviation of LRV
- ☐ Includes standard error calculation
- ☐ 95% confidence intervals of mean and/or median values
- ☐ Report the final LRV from field or laboratory (in table format)
- ☐ Report the limit of quantification
- ☐ Report the limit of detection
- ☐ Arithmetic mean pre-treatment count
- ☐ Arithmetic mean post-treatment count
- ☐ LRV limited by non-detect in product water
- ☐ LRV limited by upper detection limit
- ☐ LRV limited by lower detection limit
